# A mixed-method study on physicians’ perceptions of pay for performance: impact on professionalism, morality and work-life balance

**DOI:** 10.1186/s12913-024-12148-9

**Published:** 2025-01-14

**Authors:** Mustafa Volkan Kavas, Hasan Tut, Gamze Senyurek, Atilla Halil Elhan

**Affiliations:** 1 Department of History of Medicine and Ethics, Izmir University of Economics, Faculty of Medicine, Sakarya Cad. No:156, 35330 Izmir, Balçova Turkey; 2 Cardiology Department, Etlik City Hospital, Yenimahalle. 06170, Ankara, Turkey; 3 Department of History of Medicine and Ethics, Acibadem University, Faculty of Medicine, Kayışdağı Caddesi No:32, 34,752, İstanbul, Ataşehir Turkey; 4 Department of Biostatistics, Ankara University, Faculty of Medicine, Morfoloji Binasi, Biyoistatistik AD, 06230 Ankara, Altindag Turkey

**Keywords:** Turkey, Pay-for-performance, P4P, Estrangement, Alienation, Lived experience, Moral subject, Professionalism, Job-motivation, Job-satisfaction, Marketization, Unintended consequences

## Abstract

**Background:**

Pay-for-performance system (P4P) has been in operation in the Turkish healthcare sector since 2004. While the government defended that it encouraged healthcare professionals’ job motivation, and improved patient satisfaction by increasing efficiency and service quality, healthcare professionals have emphasized the system’s negative effects on working conditions, physicians’ trustworthiness, and cost-quality outcomes. In this study, we investigated physicians’ accounts of current working conditions, their status as a moral agent, and their professional attitudes in the context of P4P’s perceived effects on their professional, social, private, and future lives.

**Methods:**

First, we held 3 focus groups with 19 residents and 1 specialist regarding their lived experiences under P4P and thematically analyzed the transcripts. Second, we developed a questionnaire to assess how generalizable the qualitative findings are for a broader group of physicians. The tool has three parts questioning 1) demographic information, 2) working conditions, and 3) perceived consequences and effects of P4P. 2136 physicians responded to the survey. After refining the data, we conducted the statistical analysis over 1378 responses by using Spearman's correlation coefficient, exploratory factor analysis (EFA) for categorical data, and Kruskal–Wallis variance analysis.

**Results:**

Thematic analysis revealed two dimensions: 1) factors leading to estrangement, and 2) manifestations of estrangement. As for the initial, participants thought that P4P affected *relationships at work; family and social relationships; working conditions; quality of the specialty training; quality of healthcare services;* and it caused *healthcare system-related consequences*. Concerning the latter, the following themes emerged: *Estrangement of the physician*; *damaging effects on physician’s psychology*; *physician’s perception of their future life;* and *physician as a moral agent*. According to EFA, a 5-factor structure was appropriate: F1) Estrangement; F2) adverse effects on the physician’s quality of life; F3) favorable consequences; F4) physicians becoming disreputable; F5) unfavorable consequences.

**Conclusions:**

The findings suggest that under P4P, physicians have become more estranged towards their profession, their patients, and themselves. They suffer from deteriorating working conditions, lack of motivation, lack of work-related satisfaction, and hopelessness regarding their future. Furthermore, P4P impairs their ability to realize themselves as moral subjects practicing in alignment with professional values and principles.

**Supplementary Information:**

The online version contains supplementary material available at 10.1186/s12913-024-12148-9.

## Background

The Pay for Performance system (P4P), implemented in the healthcare sector for over 25 years in some Western countries, aims to enhance efficiency by subsidizing workers based on their daily productivity [1]. This system incentivizes healthcare professionals to maximize their labor capacity, leading to increased competition among individuals and ultimately higher productivity in clinical interventions [2].

While governments in numerous countries have endorsed and promoted the model in healthcare, its immediate adoption by healthcare workers has been limited. Professional bodies objected due to concerns such as increased workload, accelerated pace, erosion of regular wages, and higher rates of burnout [3–6]. Furthermore, the perceived inequity has dampened health workers' motivation, diminished interdepartmental collaboration, and strained social relations within healthcare facilities [7–9].

Parallel to the global neoliberal policies, the Turkish healthcare system underwent tremendous structural reforms over the last two decades under the Health Transformation Program (HTP)^1^ According to the Ministry of Health (MoH), HTP improved the delivery of healthcare services, increased access to healthcare, and promulgated necessary regulations for universal healthcare coverage for all citizens [10–12]. On the one hand, the provision and financing of healthcare services were disassociated, and private corporations were allowed to invest in the healthcare sector; on the other, state hospitals were autonomized financially and administratively. The former social security institutions^2^ were initially combined with each other and then transformed into a referee council, Social Security Institution (SSI – *SGK in Turkish*), that currently supervises and controls the health market on the national scale. Performance-based supplementary payment model or, in short, P4P^3^ has been one of the key elements [13, 14].

### P4P model in Turkey

In Turkey, P4P was introduced in public hospitals in 2004, extended to family physician centers in 2010, and to university hospitals in 2011, and subsequently it became integral to healthcare service delivery. The government argued that this policy aimed to enhance healthcare professionals' job motivation and elevate patient satisfaction through improved efficiency, speed, and quality of services [13, 15–18]. For that aim, physicians working under P4P received a flexible supplementary wage from institutional funds in addition to their base salaries [17, 19].

Supplementary payments to healthcare professionals are determined based on individual monthly productivity rates. Payment calculations vary among hospitals but generally depend on the number of medical interventions, such as physical examinations, diagnostic tests, and surgical operations performed by healthcare professionals [17]. Theoretically, there exists a reciprocal relationship between a hospital's financial revenue and supplementary payments to employees. Increased patient visits result in higher income for the hospital through value-added medical interventions and/or allowances from the SSI [20]. Consequently, hospitals may allocate more supplements to physicians from the circulating capital, fostering an encouragement for physicians to see more patients. The model's emergence correlates with an increased number of patients seeking medical assistance, attributed to policies promoting healthcare service consumption [3, 21–23].

Numerous sources highlight the positive impact of P4P on the success of HTP [24–27], as evidenced by increasing patient numbers, medical procedures, and users’ satisfaction rates reported in the MoH's annual healthcare reports [28]. Government officials asserted that under HTP, immediate access to physicians in public hospitals was guaranteed for all citizens [29].

Nevertheless, healthcare professionals have differing views from MoH officials regarding the impact of the P4P system. They argue that while treatment and operation quantities may increase, the quality of healthcare services declines due to worsened workplace conditions [30, 31]. Income inequity, reduced job satisfaction [32, 33], and overuse of diagnostic procedures and unnecessary or no-indication interventions are reported, leading to an increase in malpractice cases [16, 34–36]. The system also leads to limited physician–patient interaction due to the excessive number of patients [37]. High patient volumes contribute to difficulties in diagnosis, informing patients, and providing them with lifestyle guidance [34]. Accelerated working pace and competition among team members foster burnout [38] and impair open communication and solidarity among healthcare professionals, and peace at work [3, 38]. Finally, physicians and dentists expressed concerns about their inability to engage in professional development activities due to a lack of time and resources [34, 39].

### Objective

Numerous studies have demonstrated the negative effects of P4P on physicians’ working conditions and the quality of their relationships with patients and colleagues [3, 38, 40, 41]. In Turkey, increased malpractice cases, unnecessary diagnoses and treatments, rising medical costs, and the erosion of trust between patients and physicians have also been reported as consequences of the P4P system [3, 34, 35, 42]. In this study we focused on, however, how the system influences physicians’ moral accounts regarding their relationships with patients, colleagues, and families as well as their perceptions of themselves as healthcare professionals, and whether and how their professional moral attitudes changed after the implementation of P4P.

We hypothesized that P4P significantly alters healthcare professionals' work dynamics, impacting both their professional and personal lives. For this purpose, our study aimed to explore physicians' experiences as moral agents both in and outside of their workplace.

## Methods

### Design

This study focuses on examining the impact of P4P on the moral attitudes of physicians and young specialists in their daily clinical practice. Specifically, we explore whether it influences: 1) their commitment to adhering to relevant professional ethical values, codes, and norms; and 2) the working conditions that affect their ability to uphold them. Additionally, we investigate: 3) their perception of their role and responsibilities as moral agents within the context of these changes; and 4) their sentiments towards working under P4P, particularly regarding its implications for their social, personal, and future life.

Our study employs two data collection phases: Due to limited evidence regarding physicians' lived experiences under P4P, initially, we conducted focus groups (FGs) with residents from various hospitals. Subsequently, based on the findings, we designed a comprehensive questionnaire and conducted an online survey targeting physicians nationwide to assess prevalence. The rationale behind the choice of the mixed-method in the study design is to ascertain the extent to which the data obtained from the focus groups are valid for other physicians in Turkey.

Traditionally, residents bear the brunt of workload in third-stage public hospitals (university and training-research hospitals) in Turkey, primarily in outpatient services, which constitute a significant portion of healthcare institutions' revenue. As residents and young specialists are chiefly responsible for outpatient care, they are likely to be most affected by P4P. Naturally, the way they expressed their exposure to the system may have been influenced by their seniority. For instance, some of the perceived effects may be attributed to their relative lack of know-how and experience, and assumed vulnerability. Nevertheless, in our view, their perceptions constitute an upper threshold as it was thought that insights gained from this group would shed light on the perceived experiences of physicians in other categories. Therefore, to elucidate the impact of P4P on physicians through the experiences of those whom supposedly the system has affected most severely, we focused on residents and young specialists for the qualitative part of our study.

### Focus groups

In March and April 2012, we conducted 3 focus groups with 19 residents and 1 freshman specialist from 4 major institutions situated in Ankara. The participants were identified through the Ankara Medical Chamber—Resident Physician Section and through the researchers' personal networks. A total of 26 individuals agreed to participate. Two individuals subsequently cancelled, and four did not attend the scheduled session. The number of participants enrolled in each year of specialty training was as follows: two in the first year, five in the second year, nine in the third year, and three in the fourth year (Table 1). MVK, an experienced qualitative researcher, moderated the FGs. HT, HE, FA, DE and TCİ took charge of the organization and conduct of the FG sessions as assistant moderators. They were responsible both for the logistics, and the organization as well as keeping notes during the session, transcribing voice records, and archiving and analyzing the logs. All sessions were held at Ankara University Faculty of Medicine. The analysts considered the data saturation was adequate after three FGs. Except for one who was a fresh specialist in Cardiology, all the participants were residents in different fields such as General Surgery, Dermatology, Psychiatry, Hematology, Nephrology, etc.

**Table 1:** The configuration of focus groups according to the participants’ affiliation, gender and year of enrollment in specialty training

		**Focus Group No**		
		1	2	3
**Institution**	Ankara University Faculty of Medicine (8)	+	+	+
	Hacettepe University Faculty of Medicine (6)	+	+	+
	Ankara Numune Education and Research Hospital (5)	-	+	+
	Ankara Dışkapı Education and Research Hospital (1)	-	-	+
**Gender (n)**	Female	2	3	4
	Male	4	3	4
**Year of enrollment in specialty training**	1	1	1	-
	2	2	2	1
	3	3	1	5
	4	-	2	1
	1st year specialist			1
**Total (n)**		6	6	8

Informed consent to participate in the study was obtained from all the FG participants. Each session lasted approximately 1.5 h, during which we voice-recorded discussions and later transcribed them into logs. These logs and meeting minutes by assistant moderators constituted the raw data for qualitative analysis.

The moderator followed a semi-structured questioning route to inquire about participants’ thoughts and feelings. MVK and HT drafted it based on previous studies on the topic. Then two different experts from the bioethics department examined the first draft separately. One of them was a senior professor with a substantial body of work and teaching background on the physician–patient relationship and communication. The other was a relatively junior academic with experience in the field of health policy, who was a consultant to the Turkish Medical Association (TMA) at the time when we conducted the study. They gave feedback before the researchers finalized the questioning route.

The key questions are given below:

1. Has anything changed in your life with the launch of the system?
2. What is the most positive aspect of the “pay for performance” system?
3. What is the most negative aspect of the “pay for performance” system?
4. Can you describe one of your working days under the “pay for performance” system?
5. Do you think that the physicians’ relationships with their patients are affected by the “pay for performance” system?
6. Do you think that the “pay for performance” system has any effect on the relations between colleagues?
7. Have the residents’ working conditions been affected by the “pay for performance” system?
8. Has anything changed regarding your family relationships during residency?
9. Now, let’s pause for a while and imagine 3–4 years later. Where do you see yourself?

#### Analysis of focus group discussions

The researchers (MVK, HT, HE, FA, DE and TCİ) analyzed the transcripts thematically by using reflective thematic analysis approach as delineated by Braun and Clarke (2013) [43]. The researchers employed an inductive approach to analyze the qualitative data, with the objective of identifying latent themes and patterns.

Thematic analysis was selected as the most appropriate method for analyzing FGs in this study for two reasons. First, the aim is to gain insight into residents' foreseeably multidimensional experiences of P4P and to understand how these experiences affect their moral stances, and professional attitudes as well as their feelings against and their reactions to it. Second, it can reveal latent dimensions beyond this hypothetical framework.

The analysis involved several steps (Table 2): First, two researchers (MVK and HT) read each transcript independently and kept memos of possible themes (Step 1: Naïve reading/Familiarization). After a second reading, they quoted the smallest narrative units that function as answers to the key questions (Step 2: Deconstruction of the raw data). Subsequently, they coded each quote (Step 3: Coding) and categorized the emerging codes considering group dynamics—latent meanings (Step 4: Categorizing). After cross-checking their findings, they assigned themes to the categories (Step 5: Identification of the sub-themes). While coding thematically, the researchers considered frequency, specificity, emotional content, and extensiveness of participant accounts [44]. Next, they categorized the sub-themes, grouped these categories under a theme and convened consensus meetings with all researchers to finalize the themes (Step 6: Indexing). Afterwards, they tabulated contexts, themes, and sub-themes to identify a thematic framework (Step 7: Charting). The researchers then reassessed their initial analysis using these tables and reconstructed data to define patterns between contexts, themes, and sub-themes. (Step-8: Mapping). In conclusion, they interpreted the pattern structure in relation to relevant literature to gain a comprehensive understanding (Step-9: Interpretation). Finally, after consulting with the research group, they revised the report without reproaching participants for their feedback on the findings.

**Table 2:** Analysis steps

Stage	Step	Function	*Aim*
**Naïve understanding**	1	Rough reading	*Familiarization*
**Structural analysis/ Decontextualization** [considering text parts independently of their context in the text]	2	Deconstruction of the raw data	*Extracting relevant quotes*
	3	Coding	*Assigning codes to each of these units*
	4	Categorizing	*Categorization of the codes*
	5	Identifying	*Identification of the sub-themes*
	6	Indexing	*Determining the themes set*
	7	Charting	*Identification of a thematic framework*
**Comprehensive understanding/ Recontextualization** [trying to perceive the text in the light of the literature]	8	Mapping	*Reconstruction of the data: Defining the pattern of relations between contexts, themes, and sub-themes*
	9	Interpretation	*Reaching an understanding and insight*

### The questionnaire survey

Based on the FG results, the researchers created a questionnaire to quantify and assess the generalizability and validity of findings across a broader group of physicians.

Three experts (a biostatistician, a bioethicist, and a medical education expert) reviewed the initial questionnaire, leading to improvements in the second draft based on their suggestions. A pilot survey involving 40 residents from Ankara University Faculty of Medicine Hospital was conducted, leading to modifications of the tool for its final version based on preliminary results (Supplementary Material 1).

After reviewing literature on P4P effects on healthcare professionals, researchers added one more section to the final version, resulting in a tool with three sections. Section 1 consists of 8 items gathering demographic information such as age, gender, residence, title, affiliation, specialty, and marital and parental statuses. Section 2 assesses physicians' perceptions of their working conditions through 24 items divided into 5 parts: a) Perception of income, and of allocation of time for patient examination, medical interventions apart from policlinic tasks, activities for professional development, professional education, resting and relaxation, family, and social life (11 items); b) perceived workload (4 item); c) number of off-label medical practices (3 item); d) negative feelings about one’s own professional practices (3 items); and e) perceived quality of one’s communication with different parties at work (3 items). In the first part (P1), participants chose from "completely inadequate," "inadequate," and "adequate," scored 1, 2, and 3 respectively. In parts P2-5, they rated on a scale of 0–5 (0: None, 1: Quite a little/few, and 5: Too much/many). Section 3 consists of 55 items probing participants' thoughts, attitudes, and feelings regarding their experiences as professionals under P4P. Participants chose from four Likert options: "I totally disagree," "I disagree," "I agree," and "I totally agree." Twenty-one items are semantically inverse expressions.

The data were collected through haphazard sampling using an online survey software. The survey link was circulated via email lists and Facebook groups of which physicians were members. Additionally, upon the researchers' request, the Ankara Medical Chamber repeatedly called on its members to participate in the survey. In each email and Facebook message introduction, researchers kindly asked physicians to share the link with other suitable participants. The survey remained online from April 2013 to February 2018.

#### Analysis of the survey

Although the questionnaire was based on findings from FGs with residents, it was distributed to a wider range of physicians. This allowed the researchers to explore variations among physicians working in different healthcare settings and the prevalence of findings within each group.

Since the objective is to analyze the prevalence of the FG findings across the entire physician population, the sample size for each physician segment was determined based on the total number of physicians employed in Turkey in 2012 [45]. Calculations were made according to the criteria presented in Table 3 (the number of respondents in the study is also presented).

**Table 3:** Sample size and the number of respondents for the survey

Number of physicians as of 2012	Population size	Estimated true proportion	Desired precision ( ±)	Confidence level	Sample size	Number of respondents
General practitioners + Family physicians (GPs + FPs)	38.877	0.8	0.05	0.95	245	232
Residents	20.792	0.8	0.05	0.95	244	236
Specialist doctors (SDs) incl. faculty members (FMs)	70.103	0.6	0.05	0.95	368	689
Total	129.772	0.7	0.05	0.95	323	1378

2136 physicians responded to the survey, including general practitioners (GPs), family physicians (FPs), physicians in training (residents), specialist doctors (SDs), and faculty members (FMs). GPs and FPs were considered one group due to similar working conditions in Turkey. Dentists were excluded due to their distinctly variable working conditions. The institutions that participants were affiliated with were categorized as family physician centers (FPCs), training and research hospitals (TRHs), university hospitals (UHs), public hospitals (PubHs), and private hospitals (PriHs).

The data refinement process involved two criteria. First, responses lacking demographic information of age, gender, professional title, and/or institutional affiliation were removed. Second, responses with fewer than 47 items completed in Sect. 3 (< 85% response rate) were deemed invalid and excluded. This resulted in 1378 valid responses for statistical analysis.

Despite a negligible discrepancy for residents and GPs/FPs, these figures demonstrate that the number of respondents included in the study is sufficient to ensure representativeness. Furthermore, no calculations were made according to specialty, as the main variable was considered to be the health system level and institutions rather than the specialty.

Descriptive statistics included counts and percentages for categorical variables and mean and standard deviations for ordinal and continuous variables. Spearman's correlation coefficient measured variable associations. For categorical data an exploratory factor analysis (EFA) using the weighted least square method was conducted on the 55 items of Section 3 to explore the dimensionality of the item set. The Tucker Lewis Index (TLI: > 0.90 acceptable, > 0.95 excellent), the Comparative Fit Index (CFI: > 0.90 acceptable, > 0.95 excellent) and the Root Mean Square Error of Approximation (RMSEA: < 0.08 acceptable, < 0.05 excellent) were used as goodness-of-fit statistics [46]. Group differences for factors were assessed using Kruskal–Wallis variance analysis. If the *p*-value was significant, multiple comparison tests were conducted to identify differing groups. The Bonferroni correction was applied to adjust for all possible multiple comparisons. Model fit was evaluated using the root-mean square error of approximation (RMSEA) that accounts for model parsimony. RMSEA values < 0.08 suggest an adequate fit; values < 0.05 indicate a good fit [47]. Items with factor loadings below 0.30 were eliminated. Factor scores were calculated as the average of item scores. A p value less than 0.05 was considered significant.

## Results

### Findings from the FGs

The findings from the FG analyses are comprehensive. In 12 contexts, 31 themes, 82 sub-themes and numerous codes emerged. The interrelations of these elements were organized in the form of tables (Supplementary Material 2).

Following the launch of the P4P system, residents have become more estranged toward their profession, their work environment, patients, and self. Less motivation to adhere to ethical codes in the wards and loss of hope regarding the future are assumed to reinforce this tendency and heightened anxiety and burnout.

Thematic analysis uncovered dynamics contributing to physicians' perceived estrangement, which we categorize into two groups: 1) factors leading to estrangement, and 2) manifestations of estrangement.

### Factors leading to estrangement

Participants believed that P4P affected multiple facets of their lives, which we consider contributed to their estrangement. This included *relationships at work, family, and social relationships, working conditions, quality of the specialty training, quality of healthcare services, and healthcare system-related consequences of P4P*. These contexts were where critical experiences unfolded.

- 1) Relationships at work

Our analysis found that P4P negatively impacted physicians' relationships with patients, relatives, colleagues, and superiors. It hindered communication with patients and relatives, increased exposure to inappropriate behavior, and diminished perceived reputation among them.

*“. . . an anti-depressant isn’t effective in the first three weeks and in the first week it has only adverse effects. Thus, the patient takes the drug, after two days it interrupts his sleeps. . . Then, thinking that the drug prescribed by the previous one isn’t good he sees another doctor . . . He gets a second drug; he experiences the same thing until somebody finds an opportunity to explain this to him.” [FG-I/P-6/F]*^4^

Similarly, the participants viewed their superiors less favorably and felt anger towards them due to witnessing or experiencing their unethical behavior such as being treated merely as instruments.

Finally, P4P disrupted workplace peace and weakened solidarity among healthcare professionals by exacerbating relationships between physicians, teams, and departments.

*“Say, you need to go somewhere. First, you are supposed to talk to your resident fellow. Then you speak with specialists. Then, you go to your chief. That ritual is life draining. Because all say ‘sure, just go, but we are just a couple of people here.’ Once a person leaves that dirty wheel, his duties will be loaded onto someone else’s shoulder. . . [FG-III/P-5/M]*

These experiences could lead to negative feelings such as anger, anxiety, intolerance, timidity, loneliness, distrust, insecurity and reduced professional satisfaction.

- 2) Family and social relationships

Participants unanimously felt that P4P negatively impacted their family ties and narrowed their social circles, leading to receiving reduced support. Some expressed a need for understanding and support from partners or parents due to increased workload.

*“We were just married when my husband started his residency. . . The man I had known for six years turned into an utterly different person in the very first month of our marriage. It was like a nightmare . . . although you can barely keep up your own life, suddenly there is someone in need of care next to you. [FG-III/P-2/F]”*

Additionally, some expressed feeling worried or guilty for not being available when their family members needed them.

- 3) Working conditions

According to our participants, the introduction of P4P led to a surge in patient numbers, medical procedures, and administrative tasks. Hospitals suffered from understaffing, pushing participants to waive their right to vacation or leave. Additionally, they often had to handle tasks outside their job description, such as secretarial duties. Some worked overtime to meet quality standards, resulting in a heavier workload. Fear of losing performance points due to duty-offs prompted near constant work, leaving little time for rest.

*“There is a screen in between, I type there. She tells you something behind the screen. Without raising your head, you say ‘come on in, lie down’. . . Then, you look at her out of the corner of your eye and understand what it is. You run there right away and, you know, make a quick examination, then you return to your seat and slip her hand a piece of paper. You don’t really see the patient’s face, you are jammed.” [FG-I/P-5/M]*

Moreover, participants expressed frustration with direct or indirect pressure from administrators, superiors, colleagues, and the competitive drive between hospitals/departments to meet performance measures.

*“You must increase your turnovers, he said. Gosh! Is here an enterprise? What the heck are turnovers? In psychiatry, patients stay longer in hospital. Because it is necessary, . . . they already hardly collect themselves. I mean, you pull a ruined disoriented schizophrenic patient together barely in three-five weeks. But we are told to discharge them quickly in about one week! Pardon me?” [FG-I/P-1/F]*

These factors signal deteriorating working conditions, potentially leading to physician burnout, anxiety, exposure to mobbing, feelings of threat, and difficulty refreshing.

*“. . . in pediatrics residents are warned by their professor not to get pregnant. ‘If you want to conceive, show a valid reason for that, something like I am getting old (people laughing).’ I am serious, there is such a thing.” [FG-III/P-4/F]*

- 4) Quality of the residency training

Participants noted that P4P compromised the quality of residency training. Increased workload reduced time for training activities, hindered case-based learning, and limited exposure to various medical interventions of educational value, and participation in courses, and conferences. This lack of opportunities for professional development may deprive physicians of mentorship and obstruct their competence.

*“We can’t attend courses, for example. Because when somebody goes to a course, congress, or something else, the rest must do her job. That is why we are disinclined to do that. I mean, you might have to say ‘well, anyway, let me not go then.’ Then, all joking aside, four years have already been passed.” [FG-I/P-5/M]*

- 5) Quality of healthcare services

The participants stated they could not provide due care and attention to their patients under P4P due to the issues of insufficient time, tendency to commit medical errors, and automation potentially inducing feelings of incompetence and lowered self-esteem.

*“I did gastroenterology for a short time and saw 60 patients a day. . . I could not establish any communication with anybody. All I had in mind was to finish all the patients immediately, . . . I used to adopt the approach that ‘let her get a new appointment in a month [for ultrasound], and then I will not be here anyway.’” [FG-I/P-4/M]*

Additionally, they believed P4P harmed patients' health by contributing to the disruption of healthcare services, which may cause physicians to feel responsible or embarrassed about undeserved consequences.

*“Somehow, she ([the patient]) doesn’t have any other time. . . She wants to be seen even if she is the hundredth patient that day. She says, ‘I can never come again in the morning’. They ([the patients]) don’t even have the luxury to complain about this because when the quality of our lives decreases, theirs get even worse” [FG-III/P-7/M]*

Lastly, some participants suggested patients might have been pleased thinking that physicians provide better care than before.

- 6) Healthcare system-related consequences of P4P

Participants across all groups primarily highlighted negative healthcare system-related consequences of P4P. Positive effects, like enhanced service efficiency, higher physician income, and improved patient access to physicians, were mentioned but received less attention. Conversely, concerns majorly revolved around issues such as questionable diagnostic standards, impaired teamwork, service disorganization, corruption, and encouragement of unethical conduct.

*“With the diagnosis I make, this patient can’t be hospitalized. So, we change the diagnostic records. It ([the P4P regulation]) says the patient can be admitted only on that certain diagnosis. . . . We must add made-up mentions to the patient reports. We constantly play with our operation notes (Sighs).” [FG-II/P-3/M]*

The participants also discussed system malfunctions, rising healthcare costs, income disparities among professionals, physician exploitation, tarnished physician reputations, violence against physicians, and healthcare system commercialization.

*“ . . . I think they are trying to finish the preventative healthcare thing. Because there you protect the patient, and she doesn’t get sick. But there is no need to protect, let them become all ill, so that they come ([to the hospital]) and make the system run. The aim is, I mean, may money circulate, and may some people get rich.” [FG-I/P-1/F]*

Witnessing, being subjected to or taking part in multiple inappropriate, or clearly wrong practices, residents may lose faith in their profession due to feelings of stigmatization, depreciation, insecurity, and meaninglessness.

### Manifestations of estrangement

The participants specified certain dimensions of their estrangement as a central consequence of P4P. They include *the estrangement of the physician* characterized by immediate manifestations as well as the *damaging effects on physician’s psychology*, *physician’s perception of their future life* and *physician as a moral agent* albeit less directly but still significantly.

- 1) Estrangement of the physician

P4P implementations caused physicians to feel estranged from their profession, patients, others, and themselves as human beings and professionals. They expressed losing faith in and respect for the medical profession, experiencing decreased or no professional satisfaction, and feeling a gradual loss of control over vocational practices. Additionally, they voiced pessimism about the future and a reluctance to choose medicine as a profession if they could start their careers anew.

*“For example, [I say] ‘give me your hand, let’s have a look. Tongue out. Ok, done’. Because we can’t meet any medical needs. I mean, maybe not all of our training, but we can apply only a little of it. We see the patient (Participants laughing). Sometimes we see her walking, or sometimes we see her lying on the stretcher.” [FG-III/P-7/M]*

*“We perform a profession. This is not a sacred thing. Our hand is not God’s hand. All in all, it’s a profession. A job, which we do for money . . . but it’s a profession of honor, one of kind of morality. We perform a profession that all scientists performed a long time ago. . . But today, we have become a professional group whose only work is to make money.” [FG-III/P-6/M]*

Our analysis found that residents often felt angry towards their patients and relatives, displaying nervousness and intolerance when interacting with them. Moreover, they tended to extend this sentiment to the public, expressing a lack of reliance on others.

*“A patient’s relative, you ask him to leave the room because he quarrels with the nurse, and he disturbs other patients. The security guy comes to take him out. The relative says ‘What now? Should I go and call the media?’ The crud he displays is immeasurable. . . Then when the head doctor, or the chief physician, whatever, comes, ‘Oh, please, show a bit of tolerance!’ Why tolerance?!” [FG-III/P-6/M]*

The residents stated that they abstained from social interaction, preferred silence and solitude whenever feasible. Many expressed a desire to escape to uninhabited places for some time. Similarly, after a workday they could not bear conversing with or being around others and noted a declining interest in meeting friends as time went on.

*“I am worn-out, sometimes I don’t want to leave home. Let me just sit at home in the weekend, let me stand still, not go anywhere, not speak with anybody. Let me not listen to anybody’s problem. . . I long for silence. May nobody start on me for one day.” [FG-I/P-1/F]*

Residents highlighted how P4P erodes their self-confidence, compromises professional integrity, and diminishes self-esteem, citing the accelerated pace and intensity of healthcare services as factors limiting their ability to make informed clinical decisions.

*“This situation ([P4P]) . . . suppresses everything, my self-sufficiency, my self-confidence. You pull yourself back. You withdraw yourself from normal life. . . due to unnecessary workload, plus this, I mean, oppression due to the hierarchy amongst us, and the redundant work, and so on, you gradually become nonassertive surgeons. Such a surgeon is zero, I mean, nothing!” [FG-III/P-5/M]*

Finally, residents suffer from difficulty being effective in their private lives, feeling detached from reality and a sense of gradual identity loss—not being the same person they used to be. They also mentioned struggling to comprehend or process their experiences due to overwhelming workloads.

*“For the first year, it is not too abnormal that you devote all your concentration there, that you endeavor to learn, that you try to live all your days fully. But for later it turns out to be real torture. Because then you realize that everything starts to disassociate from you, you begin to live in another world and are becoming somebody else. I mean, you begin grasping more or less the place where you have ended up. You will get lonely, you will be left all alone, I mean, soon the only thing you have will be this hospital.” [FG-I/P-5/M]*

- 2) Damaging effects on physician’s psychology

Most participants noted that P4P harms their mental well-being, causing anxiety, depression, anger, disappointment, frustration, and burnout due to heavy workload, perceived injustice, and intensifying competition. This would impair physicians' quality of life and may foster indifference to misconduct and a sense of despair regarding the potential for change.

*“I get very demoralized when I see that the satisfaction I get from saving a patient’s life by sweating blood can in no sense be measured, I mean, . . . in terms of points. Perhaps, I would have earned the same number of points by merely prescribing to flu patients during that time. . . While you think that you really went over big, suddenly you realize that you have achieved nothing in terms of points.” [FG-I/P-5/M)*

- 3) Physician’s perception of their life in the future

Few participants remained hopeful about their professional future, whereas most of them grew pessimistic after P4P. This tendency seems to be originated from the fact that they anticipated negative changes and felt despair, hindering their ability to make long-term plans and driving them to constantly strive for self-improvement to avoid the unemployment risk.

*“Each and every craze of gossip, I mean, expressions such as ‘it’d be like this, like that’ make me anxious. I think it’ll never change for the better. It’s as if each upcoming day would make the running of things worse for us. After all I’ve developed anxiety of getting fresh news. I don’t want to hear anything new.” [FG-II/P-4/F]*

- 4) Physician as a moral subject

Participants suggest that P4P encourages physicians to perform or overlook unethical practices like selecting patients for higher performance points. Similarly, they prioritize performance measures over scientific algorithms when evaluating treatment indications.

*. . . in surgery, there are not many opportunities to collect points; we can’t get enough points over clinical examinations . . . Because we can only get them over surgical operations, indications have started to change, our treatments and follow-ups too. . . and that situation increases the number of complications and the surgeon’s liability. [FG-II/P-3/M]*

Residents often engage in inappropriate behaviors due to heavy workloads, such as incompliantly delegating their tasks to subordinates or seeing multiple patients simultaneously. They attribute their altered values, priorities, behaviors, and attitudes to P4P. Treating patients like customers and prioritizing quantity over quality illustrate this change.

*“I worked as a general practitioner. Back then I used to rejoice when the weather was cold, when there was a flu outbreak. That meant simple patients, making easy money. You could increase your points fabulously. For instance, while we normally had seventy patients in 24 hours, at times of the epidemic the number hit one hundred and forty. . . Although I should aim for people’s health, I rub my hands expecting them to get sick so that I can earn more money.” [FG-II/P-6/M]*

Repeatedly relying on such coping mechanisms may erode the physician's morals, leading to moral distress when they are unable to act in accordance with their professional beliefs.

*“You have no strength left to examine one more patient. It’s five to five. The one in front of you is the eightieth patient. I mean, the eightieth! . . . you had to do other things meantime, the senior professor called you over and lectured you, came down on you, etc. Now, it’s five to five. Would you examine that patient? She brought her mammography results. There are growths, you skip them unless you examine the patient.” [FG-III/P-3/M]*

### Findings from the survey

Analysis of Section 1 demonstrated the demographic attributes of the participants. 612 females (44.4%) and 766 males (55.6%) validly took part in the survey. Participants' ages ranged from 21 to 70 years, with an average of 38.6. While the majority were from major metropoles such as Ankara (n:450, 32.7%), İstanbul (n:175, 12.7%), İzmir (n:103, 7.5%), and Antalya (n:92, 6.7%), responses were received from all across Turkey (n:81). Specialist doctors (SDs) comprised the largest group, but significant data were also collected from general practitioners and family physicians (GPs + FPs), residents, and faculty members (FMs). Participants were affiliated with diverse institutions, primarily training and research hospitals (TRHs), public hospitals (PubHs), and university hospitals (UHs) (Table 4).

**Table 4:** Frequency of participants’ professional title and affiliation

		**Frequency**	**%**
**Professional title**	General practitioners and family physicians (GPs + FPs)	232	16.8
	Residents	236	17.1
	Specialist doctors (SDs)	689	50.0
	Physicians who are faculty members (FMs)	221	16.0
	Total	1378	100
**Affiliation**	Training and research hospitals (TRHs)	396	28.7
	University hospitals (UHs)	290	21.0
	Private university hospitals (PUHs)	17	1.2
	Public hospitals (PubHs)	385	27.9
	Private hospitals (PriHs)	85	6.2
	Family health centers (FHCs)	132	9.6
	Public health centers (PHCs)	17	1.2
	Other	56	4.1
	Total	1378	100

Participants represented diverse medical specialties, covering all clinical and basic branches. The largest groups were GPs and FPs (17.6%), psychiatrists (9.8%), and internal medicine specialists (5.8%). Regarding marital status, 330 (23.9%) were single, 988 (77.7%) were married, and 60 (4.4%) were divorced, with 751 (59%) having children.

Section 2 examines participants’ views on their working conditions. Items were grouped into five themes (Table 5) each with a mean value calculated. Results indicate that participants find time allocated for patient examination, non-outpatient clinic medical interventions, professional training, resting and relaxation, family and social life, and income moderately insufficient. Workload for physicians is perceived as redundant, off-label medical interventions are moderately practiced, negative feelings towards one’s own professional practices are considerably high, and the quality of communication with stakeholders is slightly below average (Table 5).

**Table 5:** Frequency of participants’ perceived evaluation of their working conditions

Part	Content (Sect. 2)	Items	Valid	Missing	$\underline{x}$±SD	Median (Min–Max)
1	Amount of income, and the time spared for patient examination, medical interventions apart from outpatient clinical tasks, professional development, professional training, resting and relaxation, family, and social life	1–11	1378	0	1.68 ± 0.44	1.64 (1–3)
2	Workload	12–15	1378	0	3.66 ± 0.90	3.75 (0–5)
3	Number of off-label medical practices	16–18	1345	33	2.24 ± 1.44	2.00 (0–5)
4	Negative feelings towards one’s own professional practices	19–21	1371	7	4.17 ± 1.03	4.67 (0–5)
5	Quality of one’s communication with different parties in daily professional life	22–24	1367	11	2.28 ± 1.12	2.33 (0–5)

In Section 3, Likert scores for the effects of P4P were: “I totally disagree” (0), “I disagree” (1), “I agree” (2), and “I totally agree” (3). Based on EFA, the six-factor solution was considered most appropriate (RMSEA = 0.046, CFI = 0.949, TLI = 0.935) with factor loadings provided in Table 6.

**Table 6:** Factor Loadings

Item	F1	F2	F3	F4	F5	F6 (Neglected)
U1	0.419					
U11	0.548					
U12	0.792					
U13	0.488					
U14	0.667					
U15	0.800					
U16	0.591					
U17	0.596					
U21	0.424					
U37	0.335					
U2		0.454				
U18		0.638				
U19		0.598				
U20		0.540				
U41		0.491				
U43		0.527				
U51		0.321				
U52		0.796				
U53		0.833				
U54		0.705				
U55		0.584				
U7			0.514			
U10			0.498			
U22			0.476			
U23			0.626			
U24			0.698			
U25			0.493			
U26			0.647			
U27			0.322			
U28			0.781			
U29			0.828			
U30			0.819			
U31			0.909			
U32			0.877			
U33			0.690			
U34			0.813			
U35			0.850			
U36			0.810			
U39			0.477			
U40			0.366			
U48			0.677			
U49			0.703			
U50			0.671			
U3				0.342		
U4				0.860		
U5				0.853		
U6				0.521		
U38					0.441	
U42					0.572	
U44					0.445	
U45					0.654	
U46					0.681	
U47					0.597	
U8						0.590
U9						0.563

One factor was neglected as only two items were loaded onto it. Cronbach’s alphas for F1, F2, F3, F4, and F5 were 0.807, 0.881, 0.918, 0.779, and 0.733, respectively. Eventually Section 3 comprises 53 items across 5 factors: F1) Professional estrangement; F2) P4P’s adverse effects on physician’s quality of life; F3) Favorable consequences of P4P; F4) Losing reputation with patients or their relatives; and F5) Unfavorable consequences of P4P (Table 7).

**Table 7:** Distribution of items (Section 3) to the factors and frequency of factor points

Factor	Content (Sect. 3)	Items	$\underline{x}$±SD	Median (IQR)
1	Professional estrangement	1, 11–17, 21, 37	2.95 ± 0.56	3.00 (1–4)
2	P4P’s adverse effects on physician’s quality of life	2, 18–20, 41, 43, 51–55	3.32 ± 0.56	3.36 (1–4)
3	Favorable consequences of P4P	7, 10^*^, 22, (23–26) ^*^, 27, (28–36) ^*^, 39, 40^*^, (48–50)^*^	3.60 ± 0.41	3.73 (1–4)
4	Losing reputation with patients or their relatives	3–6	3.49 ± 0.53	3.50 (1–4)
5	Unfavorable consequences of P4P	38, 42, 44–47	3.30 ± 0.54	3.33 (1–4)
6 (Neglected)	None	8, 9	-	-

^*^: Items coded inversely

The factor frequency analysis revealed that participants generally view P4P negatively, impacting both their professional and private lives and causing unfavorable outcomes for both professionals and the healthcare system organization. F1 scores indicated distancing from patients, a preference for easier or higher-scored medical interventions, declining faith in the profession, reduced self-confidence as a physician, and increased competition among colleagues. According to F2 results, P4P has influenced physicians’ quality of life, health, and psychology negatively and led to uncertainty, intolerance towards patients, and lack of professional satisfaction. F3 scores demonstrated participants strongly disagree that P4P brings positive outcomes such as income equity, professional security, career guarantee, work peace, healthy physician–patient relationship, efficient healthcare services, and colleague solidarity. F4 results showed that physicians link P4P and their perception of disrespect from patients and relatives, and dealing with emerging healthcare system problems alone. Lastly, F5 scores indicated a general agreement that P4P causes physicians to view patients as money or points, harms professional morals and independence, and devalues their labor (Table 7).

Differences in factor scores based on participants’ affiliation and title were analyzed. For F2 and F4, no differences were found between those working at different healthcare institutions. Concerning F1, however, physicians at PubHs felt more strongly that P4P causes professional estrangement compared to those at UHs and TRHs. In F3, physicians working at UHs have significantly higher points than those from FPCs, indicating stronger opposition to the claims that P4P has affected the healthcare organization, relationships among different parties, and physicians’ wage distribution positively. Similarly, for F5, physicians at UHs agreed more strongly than those at FPCs that P4P has impaired professional moral values and ethical practices (Table 8).

**Table 8:** Results of the factors according to participants’ affiliation

Factors	Physicians working at										P
	**Training and research hospitals (TRHs)** [*n* = 396]		**University hospitals (UHs)** [*n* = 290]		**Public hospitals (PubHs)** [*n* = 385]		**Private hospitals (PriHs)** [*n* = 85]		**Family physician centers (FPCs)** [*n* = 132]		
	$\underline{x}$± SD	Median (min–max)	$\underline{x}$± SD	Median (min–max)	$\underline{x}$± SD	Median (min–max)	$\underline{x}$± SD	Median (min–max)	$\underline{x}$± SD	Median (min–max)	
**F1**	2.88 ± 0.55^*^	2.9 (1.1–4)	2.84 ± 0.57^*^	2.9 (1–4)	3.03 ± 0.53^*^	3.1 (1.5–4)	3.05 ± 0.55	3.0 (1.7–4)	3.00 ± 0.57	3.0 (1.3–4)	< 0.001
**F2**	3.32 ± 0.53	3.4 (1.3–4)	3.26 ± 0.61	3.3 (1–4)	3.37 ± 0.56	3.5 (1–4)	3.30 ± 0.54	3.4 (1.4–4)	3.35 ± 0.54	3.4 (1–4)	0.088
**F3**	3.61 ± 0.39	3.7 (1.3–4)	3.64 ± 0.42^**^	3.8 (1–4)	3.60 ± 0.39	3.7 (1.5–4)	3.51 ± 0.46	3.6 (2–4)	3.49 ± 0.49^**^	3.7 (1–4)	0.006
**F4**	3.53 ± 0.51	3.7 (1–4)	3.44 ± 0.56	3.5 (1–4)	3.50 ± 0.52	3.5 (1–4)	3.49 ± 0.52	3.5 (2–4)	3.53 ± 0.53	3.5 (1–4)	0.218
**F5**	3.26 ± 0.53	3.3 (1–4)	3.35 ± 0.54^***^	3.5 (1–4)	3.34 ± 0.52	3.5 (1.5–4)	3.34 ± 0.50	3.5 (1.5–4)	3.18 ± 0.59^***^	3.3 (1–4)	0.004

^*^Physicians working at PubHs are different from those working at UHs and TRHs (*p* < 0.001 and *p* = 0.001 respectively)

^**^Physicians working at UHs and FPCs are different from each other (*p* = 0.009)

^***^Physicians working at UHs and FPCs are different from each other (*p* = 0.024)

Comparison by titles showed no difference in F5 scores among GPs and FPs, residents, SDs, and FMs. F1 and F2 results indicated that GPs and FPs, residents, and SDs agree more strongly than FMs that P4P has contributed to professional estrangement and diminished the quality of physicians’ lives. Residents’ F3 scores were significantly higher than the other groups, but all disapproved that P4P has improved healthcare system conduct. F4 scores showed GPs and FPs, residents, and SDs more strongly agree than FMs that P4P has discredited physicians in the eyes of patients and relatives (Table 9).

**Table 9:** Results of the factors according to participants’ title

**Factors**	**General practitioners and family physicians (GPs and FPs)** [*n* = 232]		**Physicians in training (Residents)** [*n* = 236]		**Specialist doctors (SDs)** [*n* = 689]		**Physicians who are faculty members (FMs)** [*n* = 221]		**P**
	$\underline{x}$± SD	Median (min–max)	$\underline{x}$± SD	Median (min–max)	$\underline{x}$± SD	Median (min–max)	$\underline{x}$± SD	Median (min–max)	
**F1**	3.00 ± 0.56	3.0 (1.1–4)	2.97 ± 0.54	2.9 (1.5–4)	2.99 ± 0.55	3.0 (1.5–4)	2.75 ± 0.55^*^	2.8 (1–4)	< 0.001
**F2**	3.37 ± 0.52	3.5 (1–4)	3.40 ± 0.53	3.5 (1–4)	3.34 ± 0.55	3.5 (1–4)	3.15 ± 0.60^**^	3.2 (1–4)	< 0.001
**F3**	3.55 ± 0.46	3.7 (1–4)	3.67 ± 0.38^***^	3.8 (1–4)	3.60 ± 0.40	3.7 (1.3–4)	3.55 ± 0.43	3.7 (1.2–4)	0.001
**F4**	3.56 ± 0.52	3.7 (1–4)	3.55 ± 0.48	3.8 (1–4)	3.51 ± 0.52	3.5 (1–4)	3.32 ± 0.59^****^	3.5 (1–4)	< 0.001
**F5**	3.26 ± 0.58	3.3 (1–4)	3.35 ± 0.54	3.5 (1–4)	3.32 ± 0.53	3.3 (1–4)	3.27 ± 0.52	3.3 (1.3–4)	0.203

^*^Different from GPs/FPs, residents, and SDs (*p* < 0.001, *p* = 0.001, and *p* < 0.001 respectively)

^**^Different from GPs/FPs, residents, and SDs (*p* = 0.001, *p* < 0.001, and *p* < 0.001 respectively)

^***^Different from GPs/FPs, SDs, and FMs (*p* = 0.005, *p* = 0.013, and *p* = 0.002 respectively)

^****^Different from GPs/FPs, residents, and SDs (< 0.001, *p* < 0.001, and *p* < 0.001 respectively)

Lastly, a Spearman correlation coefficient was calculated among Section 2 (perceptions about working conditions) and Section 3 (consequences and effects of P4P). Cohen’s standard was used to evaluate the strength of the relationships, where coefficients of 0.10 and 0.29 represent a small association,0.30 and 0.49 represent a moderate association, and above 0.50 indicate a large association. The results demonstrated a significant association between each part (P) of Section 2 and each factor (F) of Section 3 as follows. There is a significant negative correlation between P1 (adequacy of time and income) and F1, F2, F3, F4, and F5. The strongest correlation is between P1 and F2 (*r* = -0.51), which indicates that greater adverse effects of P4P on physicians’ lives correlate with their perception of having less adequate time for work and earning lower income. There is a significant positive correlation between P2 (perceived workload) and F1, F2, F3, F4, and F5. The correlation coefficients between P2 and F2 (0.38) and P2 and F4 (0.31) indicates a moderate relationship. This suggests that a heavier perceived workload correlates with a stronger belief that P4P adversely affects physicians. There is a significant but small positive correlation between P3 (off-label medical practices) and F1, F2, F3, F4, and F5. There is a significant positive correlation between P4 (negative feelings about professional practices) and F1, F2, F3, F4, and F5. The strong correlation with F2 (*r* = 0.59) indicates a large relationship, demonstrating that physicians’ negative feelings about their professional practices coexist with their tendency to believe that P4P adversely affects their lives. There is also a moderate association between P4 and the other factors. Lastly, there is a significant negative correlation between P5 (quality communication in professional life) and F1, F2, F3, F4, and F5. There is a moderate association between P5 and the first four factors, and a small association between P5 and F5. This indicates that as physicians perceive more negative consequences of P4P, their communication quality with colleagues, patients, and others deteriorates. Table 10 presents the results.

**Table 10:** Results of the Spearman correlation coefficient between Section 2 and Section 3

			**Section 3**				
**Section 2 / Parts**	N		**F1**	**F2**	**F3**	**F4**	**F5**
**P1 **	1378	*r*	-.331	-.506	-.414	-.412	-.310
		*p*	<0.001	<0.001	<0.001	<0.001	<0.001
**P2**	1378	*r*	.185	.378	.267	.314	.136
		*p*	<0.001	<0.001	<0.001	<0.001	<0.001
**P3**	1345	*r*	.274	.181	.142	.212	.171
		*p*	<0.001	<0.001	<0.001	<0.001	<0.001
**P4**	1371	*r*	.388	.594	.386	.427	.303
		*p*	<0.001	<0.001	<0.001	<0.001	<0.001
**P5**	1367	*r*	-.333	-.323	-.302	-.331	-.238
		*p*	<0.001	<0.001	<0.001	<0.001	<0.001

## Discussion

The results reveal multifaceted insights into physicians’ perception of their moral agency under P4P. Although closely related, we discuss the qualitative and quantitative findings separately. We believe the initial reveals the nature of physicians’ estrangement, while the latter provides insights into the underlying factors, helping us understand the role of P4P in transforming physicians to estranged professionals.

### Physician as estranged labor

FG participants explained how P4P contributed to their estrangement as physicians. By imposing a compelling form of healthcare provision, it impacts working conditions, residency training, relationships with colleagues, and family and social relationships negatively. These contextual factors reduce physicians’ self-confidence and independence and lead to anxiety and depression among them. As reported elsewhere, physicians lose their faith in their job, become indifferent to workplace issues, feel hopeless or pessimistic about the future and experience guilt or incompetency in upholding professional ethics [38, 48–50].

According to the Marxist theory of estrangement, humans can inherently use their life conditioning for willful action and consciousness. They also possess a natural ability to consciously engage with the product of their labor, crucial to their free and conscious life. Estrangement arises when humans cannot fully exercise these innate abilities [51]. As Wallimann paraphrases “... for his continued physical existence, the worker is compelled to repeatedly sell his labor power as one would sell any other commodity. But since labor power cannot in reality be separated from the locus of this power –a human being with distinct qualities and needs– the individual as the locus of labor is also treated as any other commodity.” ([51], p.27). This statement signifies that the worker waives her command over her labor power, which is nothing short of her own very self. As the worker's labor power is subjected to the owner's will, she is treated as a tool or object, serving purposes alien to her own intentions. According to Marx, “[This will result] directly in man’s estrangement from himself, from nature, from his species-being, from other men” ([51], p.96).

As seen here, estrangement has four intertwined dimensions that encompass the entire human existence. Ollmann summarizes how human nature is distorted when her essential bonds with these dimensions are broken ([52], p.133–134):

“Man is spoken of as being separated from his work (he plays no part in deciding what to do or how to do it)—a break between the individual and his life activity. Man is said to be separated from his own products (he has no control over what he makes or what becomes of it afterwards)—a break between the individual and the material world. He is also said to be separated from his fellow men (competition and class hostility has rendered to most forms of cooperation impossible)—a break between man and man. In each instance, a relation that distinguishes the human species has disappeared and its constituent elements have been recognized to appear as something else.”

With the emergence of P4P and marketization of the healthcare system in Turkey, residents seem estranged from themselves, from their professional environment, from their existential capacities, and from other people. They lose control over both the health outcomes they produce, and the way healthcare is provided.

### Physicians being estranged from themselves

Under P4P, physicians' labor is priced based on a predefined chart of medical actions they perform. Over the years, this has become the primary payment method with constant physician wages becoming comparatively trivial [38, 53]. Physicians at state hospitals earned up to six times their usual salaries in the system's early years [54], prompting them to work intensely to maintain their life standards. A survey of physicians in Turkey during the early years of P4P found that examination time per patient decreased, while the number of recorded medical tests and interventions increased [3]. These results aligned with the figures in a comprehensive 2012 MoH report [11].

FG participants stated that money has been perceived as a central measure of success in healthcare provision. Unable to meet competitive demands, they lose self-confidence, self-esteem, and struggle to maintain their dignity which could be interpreted as indicators of estrangement. Research demonstrates that physicians feel compelled to work harder than before [55–57]. Under P4P, they can maintain their standard of living only by providing healthcare services at a higher pace in an increasingly competitive market. “Producing” healthcare becomes a means, not the aim, for physicians to regain their diminishing free time for daily recovery. As their labor is involuntary and coerced, their own job has become a production process beyond their control. The authority (MoH for public hospitals, the board of trustees for private institutions), on the other hand, “determines the form of labor, its intensity, duration, the kind and number of its products, surrounding conditions and... whether or not it will even take place” ([52], p.139). By tagging each task with a point, they standardize service elements as commodities and treat physicians as tools to run the business. Consequently, physicians might have not only become estranged from their work, but also from themselves since, “[the worker’s] own active functions, his life activity, are not his but someone else’s.” ([51], p.34).

### Physicians being estranged from their professional environment

According to Marx, nature is the inorganic body of human beings with which they connect by manipulating it according to their will and consciousness to sustain their physical existence. This connection is broken if the human beings manipulate nature involuntarily for the sake of an alien will, which prevents them “from seeing, through the act of production, nature ‘as [their] work and [their] reality’” ([51], p.35).

Physicians’ work setting (nature) is composed of procedures and relationships with patients, colleagues, and administrators, influenced by the healthcare policy, organizational structure, working conditions, and rules and regulations. Today, healthcare institutions are managed like private factories due to the current trend towards marketization, and physicians -like precarious workers under capitalism- have little authority over service quality and organization. As Durán-Arenas et. al. claim “If the goals of the organization generate courses of action oriented towards economic gain, the role of physicians would be limited by the need to control costs and increase profits” ([58], p.553). Therefore, because physicians’ influence on institutional management is obstructed, they have difficulties in addressing work-related issues at a policy or legislative level [59]. In essence, they are unable to adjust their professional environment in accordance with their perspectives, possibly leading them to perceive themselves as incompetent to effect meaningful change in practice.

According to the TMA survey, P4P increased competition among healthcare professionals and reduced time for rest and off-duty activities [3]. Almost half of the physicians feel less motivated at work [3, 50, 60]. Physicians' reluctance to unite for building a collective struggle against management issues [3] may stem from their belief that they cannot change anything effectively and sustainably. It seems that under P4P, they work harder and are politically less critical about their worsening working conditions and less active to improve them [61].

Our FG participants articulated that they suffer from professional discontent and monotonous work practices. Furthermore, they expressed losing faith and respect for the profession, feeling disassociated from professional goals and their ideals gradually eroding. Many stated that they had lost control over their job, did not want to practice medicine anymore and would have not chosen to become doctors again given a second chance.

Our participants perceive their professional environment as external and self-operating, regardless of its impact on them. Marx postulates that, “the less he ([the worker]) is attracted by the nature of the work and the way in which it has to be accomplished, and the less, therefore, he enjoys it as the free play of his own physical and mental powers, the closer his attention is forced to be.” [62] According to this assertion, if physicians are hopeless and unhappy at work, they might eventually become less motivated to invest any effort in understanding and/or analyzing their working regime. In short, as moral subjects, they may become either indifferent and cynical or constantly restless due to *cognitive dissonance*^5^ arising from the inability to apply ethical codes or involuntarily being involved in unethical actions.

### Physicians being estranged from their existential capacities

Wallimann quotes Marx that “the fact the need on the part of one can be satisfied by the product of the other, and vice versa, and that the one is capable of producing the object of the other’s need, this proves... that they relate to one another as human beings; that all know their species nature to be social” ([51], p.17). Here, he highlights the existential need for mutually enriching relationships. This need arises from the fact that individuals can secure their basic interests only through human–human relationships in which they advocate for other people. Estranged labor prevents persons from reciprocally interacting, as Wallimann claims, “in such a way that ‘the need on the part of one can be satisfied by the product of the other’” ([51], p.36). Although inherently a *social being* [64, 65], a person’s essential capacities to flourish in sociality is hindered, as if she were an isolated individual. Consequently, it reduces the human being’s species-life into a means for physical existence; in other words, their social being into means for individual life. As Ollman interprets, “work has become a means to stay alive rather than life being an opportunity to do work” ([52], p.151–152).

Few studies examine how performance-based incentives affect physicians' relationships with colleagues, patients, and their families. For example, Rodriguez et al. found that patients and medical directors perceive that P4P programs result in both positive and negative outcomes. While emphasis on clinical quality and patient experience was found to be associated with improved care coordination and staff interaction, focusing on productivity and efficiency worsens physician communication and office staff interaction [66, 67]. Pertinent to this finding, Brody mentions that “when physicians are paid a lot for doing discrete, technical procedures and very little for spending time with and talking to patients, we have the sort of health system we have today, which is long on procedures and short on meaningful relationships” [68]. Furthermore, physicians expressed that professional solidarity and cooperation among healthcare staff were impaired [3, 53] which may indicate the detrimental impact of competition among colleagues [69, 70]. Under P4P, physicians are inclined to compete with other physicians for higher additional remuneration by striving to see more patients on an individual basis, rather than focusing on team-based care of fewer patients in the same time frame. They sacrifice teamwork benefits [71–74], such as work peace, joint learning, and collegiality for self-protection. Eventually, they might adopt the attitude of prioritizing personal concerns over patient care.

Competitive and insincere relationships can trigger anxiety by fostering a constant sense of threat. Correspondingly, in our study, we found that most participants suffer from insecurity and uneasiness at work due to either the unrest in the healthcare team or frequent complaints from patients. Some cited increased physician rivalry as harming solidarity among colleagues. Consequently, they described their current state as less joyful and more pessimistic, with reduced effectiveness in life. While a few participants felt detached, others struggled to comprehend their current work and living conditions.

### Physicians being estranged from other people

As Wallimann quotes from Marx “An immediate consequence of the fact that man is estranged from the product of his labour, from his life activity, from his species-nature is the *estrangement of man* from *man*. When man confronts himself, he confronts the *other* man.” ([51], p.37).

Our participants noted that under P4P, physicians might have become more disinterested in others' concerns and conflicts. Concordantly, they stated that P4P negatively affected their relationships with their family members, colleagues, and patients and relatives. Most agreed that they have become intolerant and nervous while interacting with people and feel increased anger and distrust toward others. Losing respect for peers and superiors is another common experience. Furthermore, they perceive socializing with friends and family less desirable, often preferring solitude.

The estrangement of physicians from other people can be considered most crucial since the quality of physician–patient relationship is directly related to care quality [75]. They may abstain from patients and lose interest in their unique personal stories, possibly reducing them to an abstract “patient” identity. This can eventually result in inadequate care, neglecting or deselecting patients, providing poor guidance, and not sparing sufficient time to address their concerns [76]. Moreover, they may become negligent about others' problems, leading to indifference toward individual suffering, poverty, and injustice. The estranged physician may also struggle to empathize with vulnerable groups, such as the disabled. This detachment may incline them to view patients as performance points or income opportunities.

#### Underlying contextual factors and the prevalence of physician estrangement

The survey presented the prevalence of physicians' P4P experiences and its correlations with altered working conditions. We tested our interpretations from the FGs with the survey findings, ultimately forming a holistic view of the factors leading to physician estrangement.

Demographic results showed fair gender representation. Similarly, we collected data from all parts of the country, all healthcare sectors, and all specialty branches. Most respondents were SDs, the group who, we assume, is significantly affected by P4P along with residents.

The findings suggest that physicians’ working conditions deteriorated by the implementation of P4P. For example, time for their professional development, and private and social activities diminished, and their total income became inadequate. Furthermore, most respondents have very little time for rest during workdays, and the frequency and duration of periodic leaves were very low. Additionally, they suffer from unnecessary workload, fatigue, stress, and lack of motivation and poor communication at work. Similar findings have been reported in various studies in different countries [77–81].

The EFA factors (Table 7) align with the FG thematic results. The overall factor frequencies support the study’s main premise that physicians perceive that P4P negatively affects physicians’ lives in multiple ways and contributes to the deterioration of the healthcare system.

There are slight but significant differences in factor scores by respondents' affiliation and title (Tables 8, 9). For example, PubH physicians have higher F1 scores, indicating stronger agreement that P4P contributes to professional estrangement. They might have been subjected to its influence longer due to earlier implementation at PubHs. Initially high revenues paid as incentive gradually melted down to smaller premiums as the workload increased. Moreover, physicians might have faced harsher oppression from administrators who were in charge of the initial execution of the transformation [38].

Physicians at UHs have higher F3 and F5 scores than those at FPCs, indicating stronger disagreement with the claim that P4P had positive consequences and stronger belief that P4P impairs commitment to professional ethics. These results might be related to the diverse working conditions of the two cohorts. UH physicians, working as a team, may be more affected by sharpened hierarchy, unjust wage and responsibility distribution, and worsening team relationships compared to FPs. Additionally, they might have encountered or felt compelled to apply unethical medical decisions. In contrast, FPs, working alone or leading small teams, might be more likely to overlook or engage in questionable practices due to their role in implementing P4P requirements.

FMs' lower F1 and F2 scores suggest they are less likely to believe P4P contributes to professional estrangement or diminishes physicians' quality of life. Similarly, they less strongly agree that P4P leads to disrespectful behavior from patients and their relatives (Table 9). Since the very beginning, FMs have been in a privileged position particularly in terms of income they receive. At UHs, for example, P4P widened the income gap between residents and their superiors. Payments to FMs are often based on points collected by residents and junior specialists. Similarly, it is widely believed that the payments are unequally distributed between physicians and other healthcare professionals [82–84].

Taken separately, each factor score reveals strongly shared perceptions. F1 results indicate that physicians suffer from low professional self-esteem, increased competition, and loss of faith in the profession, confirming that P4P significantly contributes to physician estrangement.

As underlying causes, the results suggest that physicians struggle to focus on time- and attention-demanding tasks, have less job satisfaction, and perceive themselves incompetent or ineffective in understanding and influencing healthcare organization and delivery. They also perceive that they have become disreputable in the eyes of patients and relatives and are exposed to increased workplace violence. Younger physicians lack adequate support from their superiors while providing healthcare services. Such experiences may cause physicians to feel victimized, worthless, and lonely. Since self-confidence refers to fulfilling one’s creative and productive potentials and effectively handling various situations [85–87], feeling professionally inactive can lead to low self-confidence and a sense of uselessness.

Performance-based incentive systems fuel competition to increase productivity [88, 89]. While competition between institutions is well-studied [90–94], its impact on individuals is often overlooked. Institutional competition pressures administrators to push physicians to work harder and at a higher pace [95, 96]; while individual competition can harm work peace, reduce solidarity, and isolate physicians [73, 97].

The results suggest that a substantial proportion of Turkish physicians have lost their faith in medicine. They feel unable to uphold their role in protecting human life and dignity due to the commercialization of healthcare, which prioritizes profit and views physicians as tools, leading to their detachment from the profession [98–101]. They probably see that P4P accelerates their detachment from the profession which can lead to despair as they must operate within the system. They may also be aware that the current state of medicine prevents them from being respected as professionals. Not being able to practice professional core values may reinforce their sense of meaninglessness.

F2 scores suggest P4P is perceived to impair physicians’ quality of professional, private and social lives. The items address uncertainty about the future, fear of losing health, affected work and family relationships, and decrease in professional satisfaction. Several studies and reports from Turkey have revealed similar findings [3, 35, 39, 57]. For example, Erdem and Atalay found that residents believe that excessive workloads due to health transformation policies severely limit time for social activities, friends, and family [39]. HTP is such a comprehensive intervention that it fundamentally redefines the foundations of the medical profession and practice. As the medical profession permeates all aspects of life, this new structure and its values may deeply influence physicians not only at work but also outside.

According to the F3 scores, most survey participants think that P4P has no favorable consequences concerning physicians’ personal rights, their relationship with patients and colleagues, and the healthcare system management. Our results align with reports demonstrating that P4P limits personal rights, creates unfair income distribution and reduces job security [50, 102]. Piece-rate payment systems are seen to harm workplace relationships, disrupt work peace and reduce solidarity among colleagues [100, 101]. It was also shown that they may harm worker health by increasing stress [103]. In Turkey, P4P has contributed to the increase in service usage and reduction in physician autonomy, raising prescription and test costs, with drug spending reaching 30% by 2009 [104]. Research on P4P's effect on service quality shows ambiguous and contradictory results. Many studies indicate P4P does not significantly improve the quality and efficiency of service delivery and, in some cases may even reduce them, contrary to what is claimed by its defendants [105–108]. In our study, most physicians did not mention any positive effect, even though a few noted that it could increase service efficiency and physician income. This view may be related to higher initial wages with the launch of the model. Nevertheless, combining our findings with similar studies suggests that P4P devalues physician labor. Even when the income decreases are not clear, the concomitant increases in working hours, workload and pace indicate physician exploitation aligning with commercialization policies that limit personal rights and professional independence.

F4 scores suggest that P4P contributed to physicians’ perception of being exposed to disrespectful behavior from patients and relatives and being burdened with healthcare system-related problems. The quality of the physician–patient relationship in Turkey has been declining over the past two decades [109]. It has been reported that physicians are being discredited publicly [110–112], as the subject of recent years' public debate, which has resulted in instances of brutal violence against them [113, 114]. This phenomenon is noted globally, especially in countries where public health practices are commercialized [115–121]. Our results are remarkable in terms of the fact that most Turkish physicians believe that P4P contributes to this issue.

According to our results, physicians lack institutional and managerial support in attempting to solve health service problems. Being seen as representatives of the healthcare sector by the public may confuse their responsibility to care for patients with obligations to run the system. Although they cannot be held responsible for its flaws, this situation may lead to service users’ unfavorable reactions toward them [122]. Working at the forefront of an unstable, problem-generating system without adequate respect for their professional competencies and institutional support when necessary, may negatively affect physicians' motivation to maintain their sense of professional integrity.

F5 scores indicate that physicians mostly agree that P4P harmed professional ethics and degraded physicians’ labor. Similarly, previous studies reported the corruption of professional values ​​and physician independence due to policies prioritizing a profitability-based productivity increase [123, 124]. Under P4P, physician labor has been morally discredited alongside a decrease in its financial value. Moreover, the current healthcare organization threatens physicians' occupational safety, professional security, right to a peaceful work environment, and the conditions required for establishing professional relationships with patients and colleagues [3, 125]. These results collectively highlight a dilemma for physicians, stemming from the irreconcilable tension between their demands for certain living and working standards and the system’s requirements that prevent these expectations from being met. As a result, physicians may abstain from interventions that could lead to ethical conflicts or obscure morally controversial cases instead of trying to resolve them. The rise in malpractice cases can be considered a reflection of this tendency [77]. In consequence, Turkish physicians may perceive themselves powerless and incompetent in terms of their “professional right-doings” potentially leading to professional dissatisfaction and depressive moods [57, 126].

Lastly, Spearman correlation analysis reveals the link between physicians' evaluations of their working conditions and their perceptions of P4P. It is well documented that the implementation of performance-based payment systems, often as a part of marketization policies, has a detrimental impact on physicians’ working conditions [40, 41, 101]. The findings suggest that challenges associated with time constraints, income adequacy, communication with patients and colleagues, and heavy workload have either been intensified with the advent of P4P, or that P4P has made physicians more vulnerable to these factors. Additionally, our respondents noted an increase in extra-regular practices such as off-label prescriptions and treatments. Similarly, it was reported that incentive systems based on performance or service might have led to an increase in malpractice cases [75, 105, 127]. Furthermore, the significant correlation between our respondents’ perceptions of P4P’s adverse effects on their lives and the feelings of lack of motivation, exhaustion, and reluctance at work highlights underlying factors contributing to their estrangement.

### Limitations

Our study has several limitations. The decision to limit the focus groups to residents, rather than including other physician groups, may be open to criticism. It was driven by two key considerations. Firstly, the budgetary and temporal constraints of the project necessitated the inclusion of only a limited number of focus groups. Secondly, given these circumstances, the decision was taken to conduct the focus groups solely with residents, as they have been the physician group hypothetically most influenced by P4P.

Additionally, the three-point scale employed in the questionnaire (Section 2), comprising the terms "completely inadequate", "inadequate" and "adequate", appears to encourage respondents to select the midpoint. Nevertheless, the section was retained in the final report. It is important to note that this section rates respondents' perceptions of working conditions and is not included in Section 3, which measures their perceptions of P4P on five factors. Despite the potential bias, we believe that the results obtained from this section should not be entirely discounted, as they offer valuable insights into respondents' contextual experiences.

It is evident that the haphazard sampling methodology raises concerns about the representativeness of the data. Nevertheless, this is a solution devised to overcome a tangible obstacle particular to the context of our country. Despite the researchers' repeated efforts, MoH obstructed collaboration with hospital administrations and withheld the number of registered physicians, their affiliations and emails from the researchers. In order to reach physicians individually, the researchers sent announcements to email groups and via active physician groups on Facebook. Despite the insufficient number of responses received, this method yielded a certain number of responses to provide a general overview. This approach was deemed the most feasible option available, as it was the only way to conduct the study.

The data collection phase of the questionnaire survey was a lengthy and intermittent process. Although we reached many physicians by this means, it can be asserted that cross-sectionality is undermined when the duration is extended and the variables that have emerged over time are disregarded. Between 2012 and 2018, the P4P system underwent considerable structural and regulatory alterations, accompanied by a notable shift in the demographic composition of the participant cohort. Nevertheless, we preferred to proceed with the study than to forgo it entirely. The researchers' personal and impersonal observations, in addition to their knowledge of the complaints of healthcare workers about the system, provided a compelling rationale for assessing the situation scientifically. Indeed, the system has been the subject of considerable controversy in Turkey for years since its inception. On the other hand, the core elements the of P4P system, such as the payment of employees on a piece-rate basis, the inadequacy of the ratio of the additional payment to the fixed salary, and the curtailment of certain personal rights, including retirement benefits remained the same. This study does not concern how the "amendments" have improved the system, but rather it examines the underlying ideology that shapes the system and its consequences for physicians. Therefore, we believe that the design and findings of our study remain valid as long as the system persists.

Another limitation is that we collected the survey data primarily through social media, including physicians' Facebook groups, to efficiently reach a substantial number of physicians. This method also helped circumvent potential obstruction by MoH in the permit application process. However, a downside of this approach is that we could not ascertain that all respondents were indeed physicians. We refined the quantitative data to overcome this constraint to a certain degree. Following a comprehensive examination of the data, we concluded it was largely reliable. Despite the limitations, our research has been the most comprehensive study on the physicians’ perceptions of P4P-related issues in Turkey. It provides insights into the causes of professional estrangement among physicians and uniquely focuses on the multidimensional transformation of physicians whose working conditions have been significantly altered by the new market-oriented payment system.

## Conclusion

In this study, we examined the effects of the P4P model on Turkish physicians. Since its implementation as part of the HTP, which restructured the healthcare system in accordance with free-market dynamics, the model has significantly altered the work regimen and redefined the fundamental aspects of health services. It has affected physicians’ professional and personal lives and prompted a shift in their moral attitudes. One significant outcome is the estrangement of physicians from their profession and inability to realize themselves as moral subjects who are supposed to practice according to professional values and principles.

In countries undergoing a similar process, physicians are portrayed by authorities and the media as the primary group responsible for the deterioration in healthcare service quality. Our findings challenge this view, showing that the quality of healthcare and physicians' adherence to professional values are linked to P4P, which multiplied their workload. The study also strongly aligns with the existing research demonstrating the negative effects of the worsening working conditions on physicians. Similarly, Turkish physicians can hardly meet their basic needs as they are deprived of full occupational security, life security, and endowment insurance. Consequently, it seems unrealistic to expect them to fully adhere to professional ethical values and principles, while ignoring the impact of marketization on healthcare workers.

Physician estrangement can be reduced or even eliminated by addressing the negative consequences of marketization in healthcare, recognizing the pressure it puts on employees and bringing these issues to the public agenda for discussion. Additionally, administrate initiatives should be devised to ensure active involvement of healthcare professionals in healthcare policymaking, improve their working conditions, raise their living standards primarily through an increase in remuneration, regulate daily patient flow in a rational manner, and minimize physicians’ exposure to competition within the healthcare setting.

## Supplementary Information

Supplementary Material 1.

Supplementary Material 2.

## Data Availability

The datasets generated and/or analyzed during the current study are available in the Open Science Framework repository: https://osf.io/v5h29/?view_only=84e69d3f3db74d43985dbeb4fab5ce79.

## Abbreviations

CFI: Comparative Fit Index
EFA: Exploratory factor analysis
F: Factor
FFS: Fee for service
FMs: Faculty members
FPCs: Family physician centers
FPs: Family physicians
FGs: Focus groups
GPs: General practitioners
HTP: Health Transformation Program
MoH: Ministry of Health
P: Part
P4P: Pay for performance system
PriHs: Private hospitals
PubHs: Public hospitals
RMSEA: Root Mean Square Error of Approximation
SSI: Social Security Institution
SDs: Specialist doctors
TDA: Turkish Dental Association
TLI: Tucker Lewis Index
TMA: Turkish Medical Association
TRHs: Training and research hospitals
UHs: University hospitals

## Footnotes

1 Health Transformation Program (HTP) is the set of policies put into practice by the government in 2003, which foresees a comprehensive change in the main dimensions of the public-based healthcare system in Turkey, such as financing, payments, organization and management, according to global free market dynamics (127).

2 Before HTP, there were three separate public social security institutions in Turkey: 1) Social Insurance Foundation *(Tr. Sosyal Sigortalar Kurumu)* for workers, 2) Retirement Fund *(Tr. Emekli Sandığı)* for civil servants, 3) Social Security Organization *(Tr. Bağkur)* for artisans and the self-employed.

3 There are notable similarities between the P4P system implemented in Turkey and the fee for service (FFS) model in the USA. Despite the Turkish model's preference for the concept of “performance” in its nomenclature, it essentially concerns the individual medical interventions performed by the physician, rather than the holistic care processes.

4 FG-I/P-6/F denotes Focus Group 1, Participant 6, Female

5 For the definition of the Cognitive Dissonance Theory please see: Festinger L (October 1962). "Cognitive dissonance". Scientific American. 207 (4): 93–102. 10.1038/scientificamerican1062-93 [63].
