# Publisher Correction: A mixed-method study on physicians’ perceptions of pay for performance: impact on professionalism, morality and work-life balance

**DOI:** 10.1186/s12913-025-12360-1

**Published:** 2025-02-21

**Authors:** Mustafa Volkan Kavas, Hasan Tut, Gamze Senyurek, Atilla Halil Elhan

**Affiliations:** 1 Department of History of Medicine and Ethics, Izmir University of Economics, Faculty of Medicine, Sakarya Cad. No:156, 35330 Izmir, Balçova Turkey; 2 Cardiology Department, Etlik City Hospital, Yenimahalle. 06170, Ankara, Turkey; 3 Department of History of Medicine and Ethics, Acibadem University, Faculty of Medicine, Kayışdağı Caddesi No:32, 34,752 İstanbul, Ataşehir Turkey; 4 Department of Biostatistics, Ankara University, Faculty of Medicine, Morfoloji Binasi, Biyoistatistik AD, 06230 Ankara, Altindag, Turkey

**Correction: BMC Health Serv Res 25, 78 (2025)**

**https://doi.org/10.1186/s12913-024–12148-9**

Following publication of the article, it was noticed that partially uncorrected page proofs were mistakenly published. The original article has been corrected and the publisher apologises to the authors and readers for the inconvenience caused by this error.

Below is an overview of the corrected errors:

**Table:** 

**Location of the correction**	**Uncorrected**	**Corrected**
Footnote 5	Citation to reference 128	Citation to reference 63
Subsections "Physicians being estranged from their existential capacities", "Physicians being estranged from other people" and “Underlying contextual factors and the prevalence of physician estrangement”	Citations to references 63-100	Citations to references 64-101
Subsection “Underlying contextual factors and the prevalence of physician estrangement”	Citation references 101, 102	Citation to references 50, 102
References" section	References 63-127	Original reference 127 renumbered to reference 63 and all subsequent references re-numbered accordingly (original reference 63 renumbered to reference 64, original reference 64 renumbered to reference 65, etc.)
Subsection “Analysis of the survey”	Calculations were made according to the criteria presented in Table 3 below (the number of respondents in the study is also presented):	Calculations were made according to the criteria presented in Table 3 (the number of respondents in the study is also presented).
Subsection “Findings from the survey”	Formatted as a level 3 heading (bold and italic emphasis)	Formatted as a level 3 heading (bold emphasis)
Reference 13	Performansa Erkan A, Ödeme Dayalı. Sağlık Bakanlığı Uygulaması. Maliye Dergisi. 2011;160:423–38. Available from: https://ms.hmb.gov.tr/uploads/2019/09/021-1.pdf. Accessed 8 Dec 2024.	Erkan A. Peformansa Dayalı Ödeme: Sağlık Bakanlığı Uygulaması. Maliye Dergisi. 2011;160:423–38. Available from: https://ms.hmb.gov.tr/uploads/2019/09/021-1.pdf. Accessed 8 Dec 2024.
Reference 22	Bostan S. What has the health transformation program in Turkey changed for patients? Hacettepe Sağlık İdaresindeki Dergisi. 2013;16(2):92–103. Available from: https://dergipark.org.tr/tr/download/article-file/84738. Accessed 8 Dec 2024.	Bostan S. What has the health transformation program in Turkey changed for patients? Hacettepe Sağlık İdaresi Dergisi. 2013;16(2):92–103. Available from: https://dergipark.org.tr/tr/download/article-file/84738. Accessed 8 Dec 2024.
Reference 31	Türk Tabipleri Birliği [Turkish Medical Association], Çankaya Belediyesi, International Association of Health Policy. Crisis of capitalism and health. In Ankara; 2011. Available from: https://www.ttb.org.tr/kutuphane/kapitalizm.pdf. Accessed 8 Dec 2024.	Türk Tabipleri Birliği [Turkish Medical Association], Çankaya Belediyesi, International Association of Health Policy. Crisis of capitalism and health. XVI. Conference of International Association of Health Policy in Europe. Ankara; 2011. Available from: https://www.ttb.org.tr/kutuphane/kapitalizm.pdf. Accessed 8 Dec 2024.
Reference 38	Kart E. The Impacts of Performance-Based Salary System as an Extension of Health Care Reforms on Physicians. Ekonomi ve Hukuk Dergisi. 2013;3(38). Available from: https://www.calismatoplum.org/makale/saglikta-donusum-surecinde-performansa-dayali-ucretlendirmenin-hekimler-uzerindeki-etkileri. Accessed 8 Dec 2024.	Kart E. The Impacts of Performance-Based Salary System as an Extension of Health Care Reforms on Physicians. Çalışma ve Toplum. 2013;3(38):104-140. Available from: https://www.calismatoplum.org/makale/saglikta-donusum-surecinde-performansa-dayali-ucretlendirmenin-hekimler-uzerindeki-etkileri. Accessed 8 Dec 2024.
Reference 44	Krueger RA, Casey MA. Focus Groups. 5th ed. SAGE; 2015. 136.	Krueger RA, Casey MA. Focus Groups: A Practical Guide for Applied Research. 5th ed. SAGE; 2015. 136.
Reference 60	Özveri O, Kayışkan K, Dağ S, Arslan B, Hisar A. Evaluation By The Physician Of The Health Performance System. Hukuk ve İktisat Araştırmaları Dergisi. 2018;10(2).	Özveri O, Kayışkan K, Dağ S, Arslan B, Hisar A. Sağkıkta Performans Sisteminin Hekimler Tarafından Değerlendirilmesi. Hukuk ve İktisat Araştırmaları Dergisi. 2018;10(2). Available from: https://dergipark.org.tr/tr/download/article-file/912519.
New reference 67	Ersahan B, Bakan I, Eyitmis A. Doctor’s negative opinions on the performance related pay. Selcuk Universitesi sosyal Bilimler Enstitusu Dergisi. 2011;(25).	Ersahan B, Bakan I, Eyitmis A. Performansa Göre Ücretlendirme’nin Doktorlar Tarafından Algılanan Negatif Yönleri. Selcuk Universitesi Sosyal Bilimler Enstitusu Dergisi. 2011;(25). Available from: https://dergipark.org.tr/tr/download/article-file/1724424.
New reference 68	New Forces Shaping the Patient-Physician Relationship. AMA Journal of Ethics. 2009;11(3):253–6. Available from: https://journalofethics.ama-assn.org/article/new-forces-shaping-patient-physician-relationship/2009-03.	Brody HA. New Forces Shaping the Patient-Physician Relationship. AMA Journal of Ethics. 2009;11(3):253–6. https://doi.org/10.1001/virtualmentor.2009.11.3.msoc1-0903.
New reference 70	van Alstyne MW. Create colleagues, not competitors. Harward Business Review. 2005;83(9):24–8.	van Alstyne MW. Create colleagues, not competitors. Harvard Business Review. 2005;83(9):24–8. Available from: https://hbr.org/2005/09/create-colleagues-not-competitors.
New reference 82	Akçakanat T. Hastanelerinde Çalışan Öğretim Üyelerinin Performansa Dayalı Ek Ödeme Sistemine Yönelik Tutumları Üzerine Bir Araştırma [A research on teaching staff?s attitudes toward performance-based payment system who are working in university hospitals]. 2013.	Akçakanat T. Hastanelerinde Çalışan Öğretim Üyelerinin Performansa Dayalı Ek Ödeme Sistemine Yönelik Tutumları Üzerine Bir Araştırma [A research on teaching staff's attitudes toward performance-based payment system who are working in university hospitals]. Dissertation. 2013. Available from: https://tez.yok.gov.tr/UlusalTezMerkezi/tezDetay.jsp?id=M0N9Veyp98ajgDIR-827hQ&no=1pzFXV-1ggpNU5sGxWD7eg.
New reference 83	Çetin AC, Sağlam H. Performansa Dayalı Döner Sermaye Prim Sistemi Uygulamasının Hizmet Sunumuna, Sağlık Çalışanlarına ve Hasta Memnuniyeti Üzerine Etkileri: Isparta Sağlık Ocaklarında Bir Araştırma. Süleyman Demirel Üniversitesi Sosyal Bilimler Enstitüsü Dergisi. 2007;5:56–78.	Çetin AC, Sağlam H. Performansa Dayalı Döner Sermaye Prim Sistemi Uygulamasının Hizmet Sunumuna, Sağlık Çalışanlarına ve Hasta Memnuniyeti Üzerine Etkileri: Isparta Sağlık Ocaklarında Bir Araştırma. Süleyman Demirel Üniversitesi Sosyal Bilimler Enstitüsü Dergisi. 2007;5:56–78. Available from: https://dergipark.org.tr/tr/download/article-file/215608.
New reference 93	Gowrisankaran G, Town RJ. Competition, Payers, and Hospital Quality1. Health Serv Res. 2003;38(6p1):1403–22. Available from: https://onlinelibrary.wiley.com/doi/10.1111/j.1475-6773.2003.00185.x.	Gowrisankaran G, Town RJ. Competition, Payers, and Hospital Quality. Health Serv Res. 2003;38(6p1):1403–22. Available from: https://onlinelibrary.wiley.com/doi/10.1111/j.1475-6773.2003.00185.x.
New reference 95	Crim RB. Exploring Health Insurance Coverage and How it is Affecting Exploring Health Insurance Coverage and How it is Affecting Patients and Providers Patients and Providers. 2021. Available from: https://egrove.olemiss.edu/hon_thesis/1857.	Crim RB. Exploring Health Insurance Coverage and How it is Affecting Patients and Providers. Dissertation. 2021. Available from: https://egrove.olemiss.edu/hon_thesis/1857.
New reference 96	Green M. Physician perceptions of productivity changes post health system employment: A mixed method case study. 2016 [Cited 2022 Jan 31]. Available from: https://www.proquest.com/openview/858f5aa23a41a1ecd3eb2c3debac0d19/1?pq-origsite=gscholar&cbl=18750&diss=y	Green M. Physician perceptions of productivity changes post health system employment: A mixed method case study. Dissertation. 2016. Available from: https://www.proquest.com/openview/858f5aa23a41a1ecd3eb2c3debac0d19/1?pqorigsite=gscholar&cbl=18750&diss=y.
New reference 97	Acar S, Sugur N. A field study on the effects of health transformation program on public employment processes. Is, Güc: Endüstri Iliskileri ve Insan Kaynaklari Dergisi. 2019;21(4):51–70.	Acar S, Sugur N. Sağlıkta Dönüşüm Programının Kamu İstihdam Süreçlerine Etkileri Üzerine Bir Alan Araştırması. Is, Güc: Endüstri Iliskileri ve Insan Kaynaklari Dergisi. 2019;21(4):51–70. Available from: https://www.isguc.org/?p=article&id=733&cilt=21&sayi=4&yil=2019.
New reference 98	Yüzden GE. Performansa Dayalı Ek Ödeme Sistemi ve Hekim Görüşlerinin Değerlendirilmesi [Performance based payment system and assessing physician’s view]. 2013.	Yüzden GE. Performansa Dayalı Ek Ödeme Sistemi ve Hekim Görüşlerinin Değerlendirilmesi. Dissertation. 2013. https://tez.yok.gov.tr/UlusalTezMerkezi/tezDetay.jsp?id=ODKObqLJbcPh5jTHkkXG8w&no=M4TuL_jzExNIgdyWa1arTg.
New reference 100	Wehkamp K-H, Naegler H. The Commercialization of Patient-Related Decision Making in Hospitals. Deutsches Ärzteblatt international. 2017; Available from: https://www.aerzteblatt.de/https://doi.org/10.3238/arztebl.2017.0797.	Wehkamp K-H, Naegler H. The Commercialization of Patient-Related Decision Making in Hospitals: A qualitative study of the perceptions of doctors and chief executive officers. Deutsches Ärzteblatt International. 2017;114:797-804. https://doi.org/10.3238/arztebl.2017.0797.
Reference 110	Bayazıt İ. Hasta-hekim ilişkisi tehdit altında. BirGün. 2020; Available from: https://www.birgun.net/haber/hasta-hekim-iliskisi-tehdit-altinda-322695.	Bayazıt İ. Hasta-hekim ilişkisi tehdit altında. BirGün. 2020; Available from: https://www.birgun.net/haber/hastahekim-iliskisi-tehdit-altinda-322695. Accessed 8 Dec 2024.
New reference 111	Ertekin A. Performance-based wage payment system and its assessment by several occupational groups in the public hospitals (Dr. Izmir. Suat Seren Chest Diseases and Thoracic Surgery Training and Research Hospital sample). 2012.	Ertekin A. Kamu hastanelerinde performansa dayalı ücret ödemesi ve çeşitli meslek grupları açısından değerlendirmesi (İzmir Dr. Suat Seren Göğüs Hastalıkları ve Cerrahisi Eğitim ve Araştırma Hastanesi örneği). Dissertation. 2012. Available from: https://tez.yok.gov.tr/UlusalTezMerkezi/tezDetay.jsp?id=lR_WtGlILzFfgi1zDG3Etw&no=FoMo0eTUTLbBJYhWGl7xnw.
New reference 117	Gopichandran V. Trust, Trustworthiness and the Physician–Patient Relationship in Developing Healthcare Settings. In 2019. p. 83–7.	Gopichandran V. Trust, Trustworthiness and the Physician–Patient Relationship in Developing Healthcare Settings. In: Dynamics of Trust in Doctor-Patient Relationship in India. SpringerBriefs in Ethics. Singapore: Springer; 2019. p 83-7. https://doi.org/10.1007/978-981-15-0346-7_7.
New reference 120	World Medical Association Condemns Attacks on Health Care Professionals. World Medical Association. 2020 [cited 2022 Jan 31]. Available from: https://www.wma.net/news-post/world-medical-association-condemns-attacks-on-health-care-professionals/.	World Medical Association Condemns Attacks on Health Care Professionals. World Medical Association. 2020. Available from: https://www.wma.net/news-post/world-medical-association-condemns-attacks-on-health-care-professionals/. Accessed 31 Jan 2022.
New reference 125	Öztürk R. Sağlıkta performansa dayalı ek ödeme sisteminin artıları ve eksileri. Medimagazin. 2013 [cited 2022 Jan 31]; Available from: https:// www.medimagazin.com.tr/dis-hekimi/tr-saglikta-performansa-dayaliek-odeme-sisteminin-artilari-ve-eksileri-3-681-52717.html.	Öztürk R. Sağlıkta performansa dayalı ek ödeme sisteminin artıları ve eksileri. Medimagazin. 2013. Available from: https://www.medimagazin.com.tr/dis-hekimi/tr-saglikta-performansa-dayali-ek-odeme-sisteminin-artilari-veeksileri-3-681-52717.html. Accessed 31 Jan 2022.
New reference 127	T.C. Sağlık Bakanlığı [Ministry of Health]. İlerleme Raporu, Türkiye Sağlıkta Dönüşüm Programı. Ankara; 2008.	T.C. Sağlık Bakanlığı [Ministry of Health]. İlerleme Raporu, Türkiye Sağlıkta Dönüşüm Programı. Ankara; 2008. Available from: https://ekutuphane.saglik.gov.tr/Ekutuphane/kitaplar/turkiyeSDP.pdf. Accessed 8 Dec 2024.

