# Supplementary material for: A mixed-method study on physicians’ perceptions of pay for performance: impact on professionalism, morality and work-life balance
Source: BMC Health Serv Res. 2025 Jan 14;25:78. doi: 10.1186/s12913-024-12148-9 (PMC11734429; doi:10.1186/s12913-024-12148-9)
Supplement: Supplementary file 1 — Supplementary Material 1. [file 12913_2024_12148_MOESM1_ESM.docx]

**QUESTIONNAIRE**

**SECTION 1 – Demographics**

1. Age: .................................
2. Gender: ( ) Male ( ) Female
3. City of residence: .................................
4. Title: .................................
5. Workplace: ( ) Training and Research Hospital

( ) University Hospital (Government)

( ) University Hospital (Private)

( ) Faculty of Medicine Basic Sciences (Government)

( ) Faculty of Medicine Basic Sciences (Private)

( ) Public Hospital

( ) Private Hospital

( ) Primary Healthcare Center

( ) Community Healthcare Center

( ) Occupational Medicine

( ) Dispensary

( ) Other (Please specify)

( ) Elderly Care Center

( ) Private Practice

1. Specialty: ……………………………………….
2. Marital status: ( ) Single ( ) Married ( ) Divorced
3. Do you have any children? ( ) Yes ( ) No

If yes, how many? …………………………**SECTION 2 – Working Conditions**

| Considering your working conditions under pay for performance system, choose the most suitable option for you. | **Completely Inadequate** | **Inadequate** | **Adequate** |
| --- | --- | --- | --- |
| 1. The duration of examination per patient |  |  |  |
| 1. Amount of time spared for tasks other than polyclinic services (i.e consultation, follow-up, etc.) |  |  |  |
| 1. Amount of time spared for scientific research |  |  |  |
| 1. Amount of time spared for educating undergraduate medical students (for academic staff) |  |  |  |
| 1. Amount of time spared for residency training (for residents) |  |  |  |
| 1. The average amount of time you spare for yourself to rest in a workday |  |  |  |
| 1. The duration of your annual leave |  |  |  |
| 1. Frequency of your leave |  |  |  |
| 1. Average time you spend with your family |  |  |  |
| 1. Amount of time you spare for your social activities |  |  |  |
| 1. Your salary/Monthly wage you receive |  |  |  |

| Considering your working conditions under pay for performance system, choose the most suitable option for you on the given scale. | *0: None*  *1: Quite little*  *5: Too much/many* |
| --- | --- |
| 1. Your average daily work hours | 0---1---2---3---4---5 |
| 1. Number of patients you see per diem | 0---1---2---3---4---5 |
| 1. Number of your night duties per month | 0---1---2---3---4---5 |
| 1. Your workload | 0---1---2---3---4---5 |
| 1. Number of out-of-indication/off-label tests in your clinic | 0---1---2---3---4---5 |
| 1. Number of out-of-indication/off-label medical interventions in your clinic | 0---1---2---3---4---5 |
| 1. Number of out-of-indication/off-label surgical operations in your clinic | 0---1---2---3---4---5 |
| 1. Reluctance to deal with your tasks | 0---1---2---3---4---5 |
| 1. Feeling of tiredness at work | 0---1---2---3---4---5 |
| 1. Work stress | 0---1---2---3---4---5 |

| Considering your working conditions under pay for performance system, choose the most suitable option for you on the given scale. | *0: Atrocious*  *1: Bad*  *5: Excellent* |
| --- | --- |
| 1. Quality of communication between you and your colleagues | 0---1---2---3---4---5 |
| 1. Quality of communication between you and patients | 0---1---2---3---4---5 |
| 1. Quality of communication between you and patient relatives | 0---1---2---3---4---5 |

| **SECTION 3 – Perceived Consequences and Effects of Pay for Performance System on physicians** |  |  |  |  |
| --- | --- | --- | --- | --- |
| Considering the pay for performance system, please choose the most suitable option for you regarding each statement. | **Totally Disagree** | **Disagree** | **Agree** | **Totally Agree** |
| 1. I always feel obliged to see more patients. |  |  |  |  |
| 1. My tolerance toward patients is decreasing gradually. |  |  |  |  |
| 1. I think only physicians are expected to solve the problems in the healthcare system. |  |  |  |  |
| 1. I am not respected enough by the patients. |  |  |  |  |
| 1. I am not respected enough by the relatives of patients. |  |  |  |  |
| 1. I think patients are provoked against physicians. |  |  |  |  |
| 1. I think pay for performance system is harmful for the physician-patient relationship. |  |  |  |  |
| 1. I think pay for performance system accelerated the work pace in clinics. |  |  |  |  |
| 1. Owing to the pay for performance system, every patient has access to a physician. |  |  |  |  |
| 1. If we did not have the pay for performance system, nobody would have worked that efficiently. |  |  |  |  |
| 1. Pay for performance system directs me to easy medical applications. |  |  |  |  |
| 1. Generally, I prioritize medical practices with higher performance points. |  |  |  |  |
| 1. I feel a pressure on my shoulders to increase the revenue of the institution I work for. |  |  |  |  |
| 1. I usually avoid complicated patients. |  |  |  |  |
| 1. Pay for performance system causes me to consider patients as points. |  |  |  |  |
| 1. I am losing my faith in my profession slowly/by time. |  |  |  |  |
| 1. I sense that I am losing my professional self-confidence as a physician. |  |  |  |  |
| 1. I have been worn down psychologically due to my professional life. |  |  |  |  |
| 1. My family life is affected negatively due to my working conditions. |  |  |  |  |
| 1. I think my future is becoming increasingly uncertain. |  |  |  |  |
| 1. After the pay for performance system competition between physicians has increased. |  |  |  |  |
| 1. I think the pay for performance system had a negative effect on in-team relationships. |  |  |  |  |
| 1. In the pay for performance system, tasks are carried out in a more planned manner. |  |  |  |  |
| 1. Thanks to the pay for performance system that my career is guaranteed. |  |  |  |  |
| 1. I am peaceful at work. |  |  |  |  |
| 1. Owing to the pay for performance system I can spend more time at home. |  |  |  |  |
| 1. I already deserve the bonus paid in the pay for performance system. |  |  |  |  |
| 1. With the pay for performance system income distribution between healthcare workers has become fair. |  |  |  |  |
| 1. Physicians feel secure under the pay for performance system. |  |  |  |  |
| 1. Pay for performance system has increased professional solidarity between physicians and non-physician healthcare personnel. |  |  |  |  |
| 1. Pay for performance system has secured the income of physicians. |  |  |  |  |
| 1. I think I will make more money in the future under the pay for performance system. |  |  |  |  |
| 1. I think scoring tests and treatments is a proper implementation. |  |  |  |  |
| 1. Without the pay for performance system nobody would have earned what they deserved. |  |  |  |  |
| 1. Pay for performance system has increased the quality of healthcare services. |  |  |  |  |
| 1. I think the pay for performance system increases the quality of specialty training. |  |  |  |  |
| 1. Sometimes I am obliged to enter false diagnoses and treatments into the database. |  |  |  |  |
| 1. Along with the pay for performance system physicians started seeing patients as money. |  |  |  |  |
| 1. I think the pay for performance system cannot assess the quality of medical interventions. |  |  |  |  |
| 1. My conscience is clear when working under this system. |  |  |  |  |
| 1. Lately I cannot get enough satisfaction from my profession. |  |  |  |  |
| 1. Pay for performance system is harmful for professional ethics. |  |  |  |  |
| 1. I am hopeless about the future. |  |  |  |  |
| 1. I think physicians do not have enough information about the pay for performance system. |  |  |  |  |
| 1. I think the pay for performance system cheapens physician labor. |  |  |  |  |
| 1. Pay for performance system obstructs physicians’ independent decision making. |  |  |  |  |
| 1. I think the details of the pay for performance system are not shared with the general public enough. |  |  |  |  |
| 1. Thanks to the pay for performance system citizens finally receive better healthcare services. |  |  |  |  |
| 1. Recently, physicians have become more respectful in the eyes of society. |  |  |  |  |
| 1. I think the pay for performance system will be improved in the future. |  |  |  |  |
| 1. If I were born again, I would have become a medical doctor again. |  |  |  |  |
| 1. I worry that I will lose my health due to my occupation. |  |  |  |  |
| 1. My quality of life is declining due to my occupation. |  |  |  |  |
| 1. Sometimes I want to distance from all people. |  |  |  |  |
| 1. I do not know where I will be in the next few years. |  |  |  |  |
