# Supplementary material for: A mixed-method study on physicians’ perceptions of pay for performance: impact on professionalism, morality and work-life balance
Source: BMC Health Serv Res. 2025 Jan 14;25:78. doi: 10.1186/s12913-024-12148-9 (PMC11734429; doi:10.1186/s12913-024-12148-9)
Supplement: Supplementary file 2 — Supplementary Material 2. [file 12913_2024_12148_MOESM2_ESM.docx]

**Supplementary Material 2: Theme tables emerged from the analysis of focus groups**

| **Context** | **Theme** | **Sub-theme** | **Code** | **Manifestation** |
| --- | --- | --- | --- | --- |
| Physician-patient relationship | Negative impacts on the physician-patient relationship | Impairment of the physician-patient communication | Feeling anger towards patients | Anger |
|  |  |  | Physician’s being nervous/intolerant in his/her relationship with patients | Intolerance |
|  |  |  | Physician’s behaving timidly his/her relationship with patients | Timidity |
|  |  | Physician’s being subjected to inappropriate behaviors | Impertinent expectations of the service recipients’ from physicians | Decrease in professional satisfaction |
|  |  |  | Being subjected to intolerance from patients |  |
|  |  |  | Being subjected to aggressive behavior from patients |  |
|  |  |  | Service recipients’ complaining about the physician (to the administration) |  |
|  |  | Disparagement of the physician | Not being respected by the patients |  |
| Physician-patient relative relationship | Negative impacts on the physician-patient relative relationship | Impairment of the physician-patient relative communication | Feeling anger towards patient relatives | Anger |
|  |  | Physician’s being subjected to inappropriate attitude | Being insulted by the patient relatives | Decrease in professional satisfaction |
|  |  |  | Not being understood by the patient relatives |  |
|  |  | Disparagement of the physician | Not being respected by the patient relatives |  |
| Superior-subordinate relationship | Negative impacts on the superior-subordinate relationship | Feeling less respect for superiors | Residents’ witnessing unethical attitude/behavior/decisions of the superiors | Derogation of the superiors |
|  |  |  | Faculty members’ losing their value in the eyes of young physicians |  |
|  |  | Feeling anger towards superiors | Superiors’ effort to cover up/ignore the tension between the physician and the patient | Isolation |
|  |  |  | Superiors’ seeing residents as instruments | Anger |
| Relationships among health professionals | Disturbance of peace at the workplace | Impairment of the relationships in the team | Feeling embarrassed before teammates | Feelings of loneliness  Feeling unsupported  Feeling under threat  Distrust  Anxiety |
|  |  |  | Feeling anger towards teammates |  |
|  |  |  | Unrest in the team because of subordinates’ earning more than superiors |  |
|  |  |  | Relationships in the team being negatively affected by increased workload |  |
|  |  |  | Cases of “stealing patients” within the department |  |
|  |  | Deterioration of the relationships among physicians | Attempt of physicians at the same institution to snatch points from each other |  |
|  |  |  | Physicians’ accusing each other of theft |  |
|  |  |  | Conflict arising from the deformation of income distribution |  |
|  |  |  | Colleagues’ being nervous at the workplace |  |
|  |  |  | Feeling less respect for the colleagues |  |
|  |  |  | Rise of distrust among colleagues |  |
|  |  | Deterioration of the relationships among health professionals | Difference of opinion among health professionals on the payment received |  |
|  |  | Deterioration of the relationships among departments | Rise of competition among different departments |  |
|  |  |  | Breakdown of the relationships between the physicians working in different departments |  |
|  |  |  | Cases of “stealing patients” among the branches |  |
|  | Impairment of the solidarity among health professionals | - | Prevention of health professionals’ acting together |  |
|  |  |  | Rise of competition among physicians |  |

Table 1: Thematic pattern of the “relationships at work” concept

| **Context** | **Theme** | **Sub-theme** | **Code** | **Manifestation** |
| --- | --- | --- | --- | --- |
| Family life of the physician | Negative impacts on family relationships | Decrease in the frequency of seeing family members | Family relationships’ fading into the background | Being destitute of social support |
|  |  |  | Physician’s not being able to spare time for his/her family |  |
|  |  | Decrease in the quality of communication with the family | Being intolerant even of the littlest things in family life |  |
|  |  |  | Overworking leading to tension in physician’s family life |  |
|  |  |  | Family relationships’ getting superficial |  |
|  | Feeling responsible to family members |  | Not being able to take care of the health of family members | Feelings of guilt |
|  |  |  | Worrying about the family members |  |
|  | Family’s adopting a supportive attitude | Need for family support | Family members understanding the exhaustion the physician experience | Needing social support |
|  |  |  | Family support providing the strength to withstand professional challenges |  |
|  |  |  | Making sacrifices for the spouse who is a physician |  |
|  |  |  | Expecting sympathy from family members |  |
|  |  |  | Family members’ showing patience |  |
| Social life of the physician | Impairment of social relationships/life | - | Shrinking of the social circle | A barren social life |
|  |  |  | Limitation of the social life due to workload |  |

Table 2: Thematic pattern of the “family and social relationships” concept

| **Context** | **Theme** | **Sub-theme** | **Code** | **Manifestation** |
| --- | --- | --- | --- | --- |
| Working conditions of the physicians | Increase in the workload of the physicians | Increase in the number of patients | Booming of the number/circulation of patients | Burnout  Not being able to refresh himself/herself |
|  |  |  | Seeing too many patients in little time |  |
|  |  |  | Administrative tendency to have all the patients see a physician |  |
|  |  |  | Patients’ seeing more than one physician for the same medical problem |  |
|  |  | Extended bureaucratic procedures | Workload caused by the obligation to enter the performed procedures into the database |  |
|  |  |  | Physician’s sparing too much time for the records of medical procedures |  |
|  |  | Understaffing | Insufficient number of health professionals other than physicians |  |
|  |  |  | Reduction of the number of health professionals in an attempt to cut the costs |  |
|  |  |  | The attempt to keep the system operating with a limited number of personnel |  |
|  |  |  | Increase in the physicians’ workload because of the personnel on leave |  |
|  |  | Being forced to perform duties that are not in the job description | Physicians’ performing secretarial tasks |  |
|  |  |  | Doing other people’s tasks all the time |  |
|  |  |  | Trying to perform a number of tasks at the same time |  |
|  |  | Worrying over losing points | Overworking to earn the optimum income |  |
|  |  |  | Not taking a leave in order not to lose points |  |
|  |  | Worrying about maintaining the quality of service | Physician’s working off-the-clock |  |
|  |  |  | Not wanting to be the person who impedes completion of the works |  |
|  |  | Increase in the number of the medical procedures performed | Increase in the number of test orders |  |
|  |  |  | Administration of unnecessary treatments |  |
|  | Lack of time |  | Having difficulties in taking a leave due to busy work | Not being able to refresh himself/herself |
|  |  |  | Loss of pay due to leave |  |
|  |  |  | Having no time left for patient examination |  |
|  |  |  | Desire to get away from the workload by becoming a specialist |  |
|  | Working under pressure | Pressure put by the administration of the institution on the physicians | Working under the pressure of seeing patients all the time | Being exposed to mobbing  Feeling under threat  Anxiety |
|  |  |  | Health institution’s encouraging the physician to prioritize procedures which would award more points |  |
|  |  |  | Physicians’ being pressured into looking out for hospital income |  |
|  |  |  | Being warned against admitting complicated patients |  |
|  |  |  | Being pressured into juggling with the figures in the database |  |
|  |  | Being subjected to bureaucratic pressure | Feeling under the pressure of prosecution for malpractice |  |
|  |  |  | Being warned against admitting the patients in need of treatment which would cause budget overrun |  |
|  |  |  | Inspection of clinics by the hospital administration |  |
|  |  |  | Hospital administration lecturing the clinics on profitability |  |
|  |  |  | Clinics being warned against making loss |  |
|  |  |  | Being pressured into standardizing the outpatient length of stay in clinics |  |
|  |  | Superiors’ pressure on the physicians | Being accused of not having worked efficiently before the performance system was introduced |  |
|  |  |  | Being pressured over performance records |  |
|  |  | Competition pressure | Different disciplines inspecting each other |  |
|  |  |  | Worrying over increasing the efficiency of the hospital |  |
|  |  | Pressure coming from the colleagues | Being pressured by the colleagues into seeing more patients |  |
|  |  |  | Exclusion of the physicians who try to act properly |  |
|  |  | Worrying over being complained about | Working under the risk of being complained about by the patients |  |

Table 3: Thematic pattern of the “working conditions” concept

| **Context** | **Theme** | **Sub-theme** | **Code** | **Manifestation** |
| --- | --- | --- | --- | --- |
| Residency training | Decline in the quality of the residency training | Residency training fading into the background | Reduction in the time spared for residency training | Not being able to develop competence |
|  |  |  | Physician’s not being able to find time to learn from his/her superiors |  |
|  |  |  | Decline in the quality of the residency training due to work overload |  |
|  |  | Decline in the quality of the case-based residency training | Not being able to see patients together with superiors (outpatient clinic) | Being destitute of the guidance of an educator |
|  |  |  | Not being able to discuss the medical cases with superiors |  |
|  |  |  | No occurrence of bedside learning/training |  |
|  |  |  | Not being able to consult superiors about the condition of patients |  |
|  |  | Physicians’ not being able to develop themselves professionally | Physician’s not being able to participate in academic meetings/professional courses | Not being able to develop competence |
|  |  |  | Not being able to see/learn varied medical practices |  |

Table 4: Thematic pattern of the “quality of the residency training” concept

| **Context** | **Theme** | **Sub-theme** | **Code** | **Manifestation** |
| --- | --- | --- | --- | --- |
| Healthcare services provided by the physician | Decline in service quality | Physician’s not being able to provide patients with proper care | Not being able to examine patients properly | Feelings of professional incompetence  Losing self-esteem in professional terms |
|  |  |  | Not being able to find time to make necessary explanations to the patient |  |
|  |  |  | Not being able to make time for the treatment the patient needs |  |
|  |  |  | Not being able to follow up his/her patients |  |
|  |  | Physician’s not being able to pay proper attention to patients | Not being able to listen to the patients properly |  |
|  |  |  | Not being able to provide life style guidance to the patients |  |
|  |  |  | Not being able to meet the basic expectations of patients |  |
|  |  |  | Ignoring the differences between patients |  |
|  |  |  | Examining more than one patient in the outpatient clinic |  |
|  |  | Physician’s not being able to practice his/her profession properly | Automation in service provision |  |
|  |  |  | Making medical errors more frequently |  |
|  |  |  | Excessive number of requests for consultation |  |
|  |  |  | Decrease in time allocated by the physician to each patient |  |
| Healthcare services received by the patients | Decline in service quality | Negative impacts on patients’ health | Delayed/late diagnosis | Feeling responsible to patients  Feeling embarrassed |
|  |  |  | Failure to follow basic medical procedures (Physical examination, anamnesis) |  |
|  |  |  | Delayed recovery process |  |
|  |  |  | Some patients’ being deprived of treatment due to package overrun |  |
|  |  |  | Difficulties in accessing the physician to ask for advice |  |
|  |  |  | Negative effects of the decline in the physicians’ quality of life on patients |  |
|  | Positive opinions of the patients | - | Patients’ thinking that they receive better care than before | Professional satisfaction |

Table 5: Thematic pattern of the “quality of healthcare services” concept

| **Context** | **Theme** | **Sub-theme** | **Code** | **Manifestation** |
| --- | --- | --- | --- | --- |
| Effects of the performance system | Positive aspects of the performance system | Improvement in the efficiency of healthcare services | Acceleration of the service-related processes | Neutral |
|  |  |  | Making those who do not want to work work | Professional satisfaction |
|  |  | Income growth | The idea that the performance system increases the income |  |
|  |  | Increased access to physicians | Increase in patient access to physicians |  |
|  | Negative aspects of the performance system | Decline in the quality of healthcare services due to standardized diagnoses | The fact that the required medical procedures cannot be performed due to limited insurance coverage | Witnessing unruliness |
|  |  |  | Difficulty in standardizing the tests requested |  |
|  |  | Negative impacts on teamwork | Disruption of the coordination among departments |  |
|  |  |  | Disruption of team functioning due to exhaustion | Getting tired unnecessarily |
|  |  | Problems with time management | Decrease in the examination time due to entering the procedures into the database |  |
|  |  | Disruption of the organization of healthcare services | Disruption of a well-functioning system for the sake of unearned income | Witnessing unfairness  Witnessing unruliness |
|  |  |  | Disruption of the functioning staff organization |  |
|  |  |  | Arbitrary/unruly behaviors of the new administration |  |
|  |  |  | Defunctionalization of the competent staff |  |
|  |  | Corruption | Disregard for merits |  |
|  |  |  | Decline in the quality of healthcare services |  |
|  |  |  | Flouting of ethical values in healthcare settings | Witnessing unethical behaviors  Being a part of unethical practices |
|  |  | Encouragement of inappropriate behaviors by the system | Increased rate of record-keeping violations |  |
|  |  |  | Prompting people to engage in wrong behaviors |  |
|  |  |  | Being encouraged to resort to effortless practices |  |
|  |  |  | Being encouraged to resort to practices that would award points |  |
|  |  | Dysfunctioning of the healthcare system | The healthcare system providing no benefit for anyone | Sense of meaninglessness |
|  |  |  | Decline in the efficiency of healthcare services |  |
|  |  | Increase in healthcare costs | Emergence of the need for extra labor force in healthcare |  |
|  |  | Deformation of income distribution among health professionals | Underpayment to health professionals’ other than physicians | Witnessing unfairness |
|  |  |  | Unfair payments when compared to other occupational groups |  |
|  |  | Unfairness of the salary system | The disproportion between the heavy working conditions and the amount paid in return | Being subjected to unfairness |
|  |  |  | Preference of adequate and fixed income over additional payments |  |
|  |  |  | No increase in the total amount paid to the physicians |  |
|  |  |  | Additional payments’ having no effect on pension receivables |  |
|  |  |  | Insufficient fees assigned for each unit of practice |  |
|  |  |  | One not being fairly paid in return for his/her work |  |
|  |  |  | Decrease in additional payments in the course of time |  |
|  |  |  | Inadequacy of physician salaries |  |
|  |  |  | Thinking that the payment being received is already well-deserved |  |
|  |  | Physicians’ turning into laborers | Residents’ turning into cheap labor | Being subjected to exploitation |
|  |  |  | Interns being considered as cheap labor |  |
|  |  |  | Cheapening of the physician’s labor |  |
|  |  |  | Impoverishment of the physicians |  |
|  |  |  | Melting down of the physician salaries |  |
|  |  | Disparagement of the physicians | Physicians’ being looked down upon as potential imposters | Being labeled  Derogation |
|  |  |  | Physicians’ losing their social status |  |
|  |  |  | Politicians’ not respecting physicians |  |
|  |  |  | Politicians’ disparaging the physicians |  |
|  |  |  | The profession losing its reputation in the society |  |
|  |  |  | Repudiation of the effort put in by the physician |  |
|  |  | Violence against physicians | Threats/insults directed by patient relatives at physicians | Being concerned about the security of life |
|  |  |  | The fact that the system enables the service recipients to let out their accumulated rage at physicians |  |
|  |  |  | Physicians’ being pointed as targets by the politicians |  |
|  |  | Commercialization of healthcare | Commercialization of the service provision | Sense of meaninglessness |
|  |  |  | The fact that professional success is measured by the profit generated for the institution |  |
|  |  |  | Evaluation of the profession merely with reference to money |  |
|  |  |  | Physicians’ being evaluated over the revenue they generate for the institution they work for | Being exploited/ Derogation |
|  |  | Paradigm shift in the provision of healthcare services | Performance system dictating the services to be provided | Sense of meaninglessness |
|  |  |  | The fact that the points have become the assessment criterion for service quality |  |
|  |  |  | Shifting professional priorities towards gaining more points |  |
|  |  |  | Money becoming a key determinant in healthcare service |  |
|  |  |  | Disintegration of the tradition of care |  |
|  |  | Distortion of the facts about performance system | Failure to offer service recipients an insight into the performance system | Facing false accusations |
|  |  |  | Misinformation of public about the remuneration process |  |
|  |  | The idea that physicians are mispresented to the society | Fostering the idea that the unfavorable situations are caused by physicians |  |
|  |  |  | Accusation of physicians of being money-grubber |  |
|  |  |  | Misrepresentation of the physicians’ working conditions to the public |  |
|  |  |  | Placing the burden of the problems created by the system upon physicians |  |
|  |  | The system pushing people into competition | Encouragement of competition by the system | Being subjected to exploitation |
|  |  |  | Competition among individuals due to unfair distribution of income |  |
|  |  | Thinking that the system is wrong | Finding it wrong that the additional payments exceed the base salary | Witnessing unfairness |
|  |  |  | The fact that the performance system is based upon unfair distribution of income |  |
|  |  |  | The fact that the system has been launched without a well-conceived plan | Being a part of wrong practices |
|  |  |  | The fact that the system has been introduced as part of a populist agenda |  |
|  |  | Not believing in the parameters of the performance system | Unfairness of the standards of the scoring |  |
|  |  |  | Not scoring the good medical practice |  |
|  |  |  | Not measuring the quality of healthcare services |  |
|  |  | Breakdown of solidarity among physicians | Absence of struggle for the rights lost | Loneliness |
|  |  | Rise of indifference among physicians | Insensitivity to existing problems | Cynicism |
|  |  |  | Depoliticization |  |
|  |  | Physicians’ expressing their objections | Physicians’ not being able to have a say in the decision-making process related to their profession | Solution seeking |

Table 6: Thematic pattern of the “healthcare-system related consequences” concept

| **Context** | **Theme** | **Sub-theme** | **Code** | **Manifestation** |
| --- | --- | --- | --- | --- |
| Estrangement of the physician | Changes in the physician’s perception of the profession | Physician’s estrangement from his/her profession | Decrease in the physician’s faith in his/her profession | Physician’s estrangement from the production process |
|  |  |  | Decrease in the physician’s respect for the profession |  |
|  |  |  | Erosion/shaking/breaking down of the professional ideals |  |
|  |  |  | Professional practices’ becoming monotonous |  |
|  |  |  | Professional dissatisfaction |  |
|  |  |  | Losing control over the profession as a physician |  |
|  |  |  | Estrangement from the objectives of the profession |  |
|  |  |  | Pessimism about the future of the profession |  |
|  |  |  | Not wanting to be a physician/not wanting to practice medicine anymore |  |
|  | Changes in the physician’s perception of other people | Physician’s estrangement from the patients | Anger towards the public | Physician’s estrangement from human relationships |
|  |  |  | Distrust in the public |  |
|  |  |  | Feelings of anger towards patients |  |
|  |  |  | Physician’s being nervous/intolerant in his/her relationship with the patient |  |
|  |  | Physician’s estrangement from other people | Decrease in the desire to meet friends |  |
|  |  |  | Desire to be alone |  |
|  |  |  | Quest for silence |  |
|  | Changes in the physician’s perception of his/her self | Physician’s estrangement from himself/herself as a professional | Physician’s losing self-confidence | Physician’s estrangement from his/her own labor process |
|  |  |  | Physician’s losing self-esteem |  |
|  |  |  | Physician’s not being able to protect his/her dignity |  |
|  |  |  | Not being able to meet professional requirements |  |
|  |  | Physician’s estrangement from himself/herself as a human being | The feeling of not being effective in his/her own life | Physician’s estrangement from his/her own existence |
|  |  |  | Not being the same person anymore/Being transformed |  |
|  |  |  | Not having a life outside of work/Detachment from life |  |
|  |  |  | Not being able to interpret what one is going through due to work overload |  |
|  |  |  | Not being able to develop consciousness due to work overload |  |

Table 7: Thematic pattern of the “Estrangement of the physician” concept

| **Context** | **Theme** | **Sub-theme** | **Code** | **Manifestation** |
| --- | --- | --- | --- | --- |
| Psychology of the physician | Negative impacts on the physician’s mental health | Increase in professional anxiety | The fact that the possibility of overrunning the package causes stress | Decrease in the quality of life of the physician |
|  |  |  | Fear of being complained about |  |
|  |  |  | Experiencing work-related anxiety on days off |  |
|  |  |  | Anxiety arising from the competition among the physicians |  |
|  |  |  | Physicians’ experiencing anxiety about maintaining their lives |  |
|  |  |  | Breaking of the peaceful atmosphere at the workplace |  |
|  |  |  | The anxiety of feeling that anything can change at any time |  |
|  |  |  | Not being able to resign due to fear of insecurity |  |
|  |  | Burnout | Physicians’ not being able to find time rest on workdays |  |
|  |  |  | Forgetting/failing to follow routine/daily activities |  |
|  |  |  | Physician’s not being able to meet his/her basic needs due to work |  |
|  |  |  | Weariness arising from solving problems ceaselessly |  |
|  |  |  | Feeling of suffocation due to the fact that everyone has a demand from the physician |  |
|  |  |  | Becoming a more angry/intolerant person |  |
|  |  |  | Physicians’ not being able to maintain the part of his/her life which falls outside of work |  |
|  |  |  | Not taking a leave in order not to lose points |  |
|  |  | Depressive mood | Not being able to enjoy life |  |
|  |  |  | Desire to keep away from people |  |
|  |  |  | Not wanting to see people |  |
|  |  |  | Getting bored of people |  |
|  |  |  | Pessimism |  |
|  |  | Anger | Feeling anger due to having to perform duties that are not in the job description |  |
|  |  | Disappointment | Not being able to achieve professional ideals |  |
|  |  |  | Disappointment due to unfair income distribution |  |
|  |  |  | Disappointment about the expectation of a comfortable life |  |
|  |  | Frustration | Frustration due to misinformation of public about the payment that the physicians receive |  |
|  |  | Indifference/Nonchalance | Indifference towards unfavorable situations/wrongdoings | Becoming cynical |
|  |  |  | Nonchalance due to having to perform duties that are not in the job description |  |
|  |  |  | Physicians’ accommodating themselves to unfavorable situations |  |
|  |  |  | Desire to shift the responsibility off |  |

Table 8: Thematic pattern of the “Detrimental effects on physician’s psychology” concept

| **Context** | **Theme** | **Sub-theme** | **Code** | **Manifestation** |
| --- | --- | --- | --- | --- |
| Physician’s perceptions of the life in the future | Uncertainty about the future | Not being able to make any predictions about the future | Not being able to make long-term plans | Uncertainty |
|  |  |  | Thinking that there is a risk of unemployment in the future | Anxiety |
|  |  |  | Feeling an urge to improve oneself continuously not to be unemployed |  |
|  | Pessimism about the future | Thinking that the future changes will be negative | Thinking of changing/leaving the job in the future | Pessimism |
|  |  |  | Prediction of being like the people they are criticizing now in the future |  |
|  |  |  | Thinking that the healthcare institutions will be privatized in the future |  |
|  |  |  | Being afraid of having to live away from the family and friends in the future | Anxiety |
|  |  |  | Not wanting to receive news of any more changes |  |
|  |  | Feelings of despair about the future | Desire to flee abroad in the future | Hopelessness |
|  |  |  | Not having the hope that things will change in the future |  |
|  | Hope that things will change in the future | - | Desire to work in an isolated working environment in the future | Hope |
|  |  |  | Enduring present hardships with the hope of being at ease in the future |  |

Table 9: Thematic pattern of the “Physician’s perception of their life in the future” concept

| **Context** | **Theme** | **Sub-theme** | **Code** | **Manifestation** |
| --- | --- | --- | --- | --- |
| Case of the physician as a moral subject | Drifting away from professionalism | System’s prompting the physician to engage in unethical conduct | Entering the procedures that have not been performed into the database | Not being able to put/not putting ethical codes into practice |
|  |  |  | Preferring the tests/treatments awarding more points in a shorter time |  |
|  |  |  | Picking the patients with higher points |  |
|  |  |  | Failure to protect/violation of patient confidentiality |  |
|  |  |  | Taking over the patients that he/she did not examine on the system |  |
|  |  |  | Meeting inappropriate demands of the patients |  |
|  |  |  | Performing off-label interventions |  |
|  |  | Performance system’s making it hard to comply with scientific criteria | Flexing the scientific standards for the sake of collecting points |  |
|  |  |  | Taking performance points into consideration in indication evaluations |  |
|  |  | Physician’s displaying inappropriate behavior to cope with work overload | Physician’s having to ask subordinates to carry out the works he/she does not believe in |  |
|  |  |  | Examining more than one patient at the same place (outpatient clinic) |  |
|  |  |  | Physicians’ performing interventions for which they are not competent enough |  |
|  |  |  | Not being able to find time to report the problems witnessed |  |
|  |  | Changes in the attitude and behavior of the physician | Physician’s behaving like a shopkeeper who tries to please his customer in his/her relationship with the patient |  |
|  |  |  | Physician’s making money over the patient |  |
|  |  | Changes in the values/priorities of the physician | Caring more about the money to be earned than the quality of the service | Moral erosion |
|  |  |  | Money taking precedence over the wellbeing/caring of the patient |  |
|  |  |  | Seeing patients as points/money |  |
|  |  |  | Justifying inappropriate conducts people engage in to gain more points |  |
|  |  |  | Physicians’ desire to make more money by seeing more patients |  |
|  | Cognitive dissonance | Physician’s feeling the weight of not being able to do what is professionally right on his/her conscience | Feeling bad due to being a part of wrong practices | Moral burden of behaving in a way that he/she finds unethical |
|  |  |  | Feeling bad due to making false declarations |  |
|  |  |  | Not being able to practice medicine with dignity |  |
|  |  |  | Thinking that patients get harmed due to the off-label interventions |  |
|  |  |  | Being caught in between taking proper care of the patients and gaining more points |  |
|  |  |  | Giving up on his/her own principles |  |

Table 10: Thematic pattern of the “Physician as a moral subject” concept
